# The impact of home-based management of malaria on clinical outcomes in sub-Saharan African populations: a systematic review and meta-analysis

**DOI:** 10.1186/s41182-023-00572-2

**Published:** 2024-01-08

**Authors:** Kok Pim Kua, Shaun Wen Huey Lee, Bunchai Chongmelaxme

**Affiliations:** 1 Department of Civil and Environmental Engineering, School of Engineering and Doerr School of Sustainability, Stanford University, Stanford, CA 94305 USA; 2 MIT Alumni Association, Massachusetts Institute of Technology, Cambridge, MA 02139-4822 USA; 3 Pharmacy Unit, Puchong Health Clinic, Petaling District Health Office, Ministry of Health Malaysia, 47100 Puchong, Selangor Malaysia; 4 A.S. Watson Group, Watson’s Personal Care Stores, 55188 Kuala Lumpur, Malaysia; 5 School of Pharmacy, Monash University, Bandar Sunway, 47500 Subang Jaya, Selangor Malaysia; 6 Asian Center for Evidence Synthesis in Population, Implementation, and Clinical Outcomes (PICO), Health and Well-Being Cluster, Global Asia in the 21st Century (GA21) Platform, Monash University, Bandar Sunway, 47500 Subang Jaya, Selangor Malaysia; 7 Gerontechnology Laboratory, Global Asia in the 21st Century (GA21) Platform, Monash University, Bandar Sunway, 47500 Subang Jaya, Selangor Malaysia; 8 Faculty of Health and Medical Sciences, Taylor’s University, Subang Jaya, 47500 Lakeside CampusSelangor, Malaysia; 9 Center for Global Health, Perelman School of Medicine, University of Pennsylvania, Philadelphia, PA 19104 USA; 10 Department of Social and Administrative Pharmacy, Faculty of Pharmaceutical Sciences, Chulalongkorn University, 254 Phayathai Road, Patumwan, Bangkok, 10330 Thailand

**Keywords:** Malaria, Antimalarial, *Plasmodium falciparum*, Home management, Home delivery, Artemisinin-based combination, Community health worker, Malaria eradication, Malaria control, Control and elimination programmes, Control and elimination interventions

## Abstract

**Background:**

Malaria remains a significant cause of morbidity and mortality globally and continues to disproportionately afflict the African population. We aimed to evaluate the effect of home management of malaria intervention on health outcomes.

**Methods:**

In our systematic review and meta-analysis, six databases (Pubmed, Cochrane CENTRAL, EMBASE, CAB Abstracts and Global Health, CINAHL Complete, and BIOSIS) were searched for studies of home management of malaria from inception until November 15, 2023. We included before–after studies, observational studies, and randomised controlled trials of home management intervention delivered in community settings. The primary outcomes were malaria mortality and all-cause mortality. The risk of bias in individual observational studies was assessed using the ROBINS-I tool, whilst randomised controlled trials were judged using a revised Cochrane risk of bias tool and cluster-randomised controlled trials were evaluated using an adapted Cochrane risk of bias tool for cluster-randomised trials. We computed risk ratios with accompanying 95% confidence intervals for health-related outcomes reported in the studies and subsequently pooled the results by using a random-effects model (DerSimonian–Laird method).

**Results:**

We identified 1203 citations through database and hand searches, from which 56 articles from 47 studies encompassing 234,002 participants were included in the systematic review. All studies were conducted in people living in sub-Saharan Africa and were rated to have a low or moderate risk of bias. Pooled analyses showed that mortality rates due to malaria (RR = 0.40, 95% CI = 0.29–0.54, *P* = 0.00001, *I*^2^ = 0%) and all-cause mortality rates (RR = 0.62, 95% CI = 0.53–0.72, *P* = 0.00001, *I*^2^ = 0%) were significantly lower among participants receiving home management intervention compared to the control group. However, in children under 5 years of age, there was no significant difference in mortality rates before and after implementation of home management of malaria. In terms of secondary outcomes, home management of malaria was associated with a reduction in the risk of febrile episodes (RR = 1.27, 95% CI = 1.09–1.47, *P* = 0.002, *I*^2^ = 97%) and higher effective rates of antimalarial treatments (RR = 2.72, 95% CI = 1.90–3.88, *P* < 0.00001, *I*^2^ = 96%) compared to standard care. Home malaria management combined with intermittent preventive treatment showed a significantly lower incidence risk of malaria than home management intervention that exclusively provided treatment to individuals with febrile illness suggestive of malaria. The risks for adverse events were found to be similar for home management intervention using different antimalarial drugs. Cost-effectiveness findings depicted that home malaria management merited special preferential scale-up.

**Conclusions:**

Home management of malaria intervention was associated with significant reductions in malaria mortality and all-cause mortality. The intervention could help decrease health and economic burden attributable to malaria. Further clinical studies are warranted to enable more meaningful interpretations with regard to wide-scale implementation of the intervention, settings of differing transmission intensity, and new antimalarial drugs.

**Supplementary Information:**

The online version contains supplementary material available at 10.1186/s41182-023-00572-2.

## Background

Malaria is a devastating infectious disease that is a major cause of morbidity and mortality [1], with sub-Saharan Africa shouldering the heaviest burden [2]. Estimates show that there were 241 million malaria cases and 627,000 malaria-related deaths across the globe in 2020, an increase between 7 and 12% compared to 2015 [3], of which, the African region accounts for 95% of the world's malaria cases and 96% of mortality [2]. As such, prompt and effective treatment of malaria is critical in preventing progression to severe disease or complications and reducing morbidity and mortality. The increasing resistance to chloroquine has led to the use of artemisinin-based combination therapy as the first-line treatment against confirmed or suspected *Plasmodium falciparum* malaria [4, 5]. Alongside a functioning continuum of care that encompasses recognition of severe episodes at household and primary care levels to prompt comprehensive management with effective diagnostics and medicines, the risk of death or permanent disability could be remarkably reduced [6].

Home-based management of malaria has been promoted as a strategy to increase early diagnosis of malaria, physical access to antimalarial drugs, and use of malaria preventive treatment [7]. It is recommended under the Roll Back Malaria Initiative to reduce the burden of malaria by delivering effective antimalarial treatment to individuals with suspected malaria so that they can receive appropriate care in the comfort of their own homes [8, 9]. It encompasses components, such as establishment of a suitable platform that empowers caregivers to recognise malarial illness early and respond accordingly, community-based training programmes that equip caregivers with adequate knowledge and capacity to respond to malarial illness, and creation of an environment that facilitates the provision of antimalarial drugs as close as possible to the patients’ homes [8, 10]. As such, community health workers and community drug distributors are instrumental in implementing the home management intervention [10]. However, home management of malaria is associated with several potential disadvantages, such as unnecessary overtreatment, emergence of drug resistance, and higher costs [11]. In recent years, there have been home-based management of malaria programmes that integrate diagnosis and treatment based on test results, leading to a better community access to prompt and effective management of uncomplicated malaria, particularly in rural areas with high levels of transmission [12, 13].

Home-based malaria management intervention plays a vital role to ensure the reach of public health services, particularly for populations living in rural, remote, or hard-to-reach areas, where there is limited access to healthcare infrastructures. It allows proactive case detection and treatment via the scaling up of integrated community delivery platforms and supports a broader continuum of care for impoverished groups who often face disadvantage, discrimination and exclusion in health outcomes. Such intervention that is tailored to the local context accelerates the progress along the path to control and ultimately eliminate malaria. In line with a global effort to eradicate malaria, all suspected malaria cases should receive timely diagnostic confirmation through parasite detection methods, such as antigen-detection rapid diagnostic tests and microscopy examination of blood films. Home-based management of malaria enhances the access to prompt diagnostic testing in remote sites and contributes to effective treatment of uncomplicated malaria or other medical conditions. It alleviates the burden of preventable and treatable deaths and diminishes the risk of onward transmission of malaria in the communities [14].

Home-based management of malaria (HMM) is a promising strategy to improve public access to prompt and effective management of uncomplicated malaria for reducing the disease burden. It encompasses the provision of diagnostic tests and pharmacological therapies to patients close to their homes [12, 15]. Community case management of malaria (CCM) is designed to reach a larger fraction of the population by bringing primary health care into the communities. It involves the training of community health workers to diagnose and treat uncomplicated malaria cases within their communities [16]. On the other hand, home-based management of malaria (PECADOM) is targeted for inhabitants of remote or rural areas with difficulties in accessing health care where community health workers visit all households in their communities weekly during malaria transmission season to identify fever cases and offer case management [17]. Community Health Workers (CHWs) are trusted members of local communities who are trained to play a bridging role between patients and clinical services, transferring and interpreting health information and ensuring that patients are connected to health care [18, 19].

The impact of home management intervention on malaria morbidity and mortality has been inconclusive, with existing research depicting mixed findings [8]. To date, there is only one systematic review of home-based treatment of malaria which is limited to the use of chloroquine alone and it was found that the evidence base on home management of malaria intervention were sparse and showed inconsistent results [11]. An updated systematic review is necessary to expand the former literature searches in view of the drug resistance to chloroquine and new standard of care with artemisinin-based combination therapy for malaria [20, 21]. Therefore, the aim of the current review was to provide a comprehensive overview on the effect of home-based management of malaria and its impact on health outcomes.

## Methods

This systematic review with meta-analysis was conducted by following the PRISMA (Preferred Reporting Items for Systematic Review and Meta-Analyses) guidelines, 2020 [22].

### Search strategy and selection criteria

Six online bibliographic databases were searched from inception up to November 15, 2023: Pubmed, Cochrane CENTRAL, EMBASE, CAB Abstracts and Global Health, CINAHL Complete, and BIOSIS for studies that investigated the effect of home management of malaria. No restrictions on language, age, geography, document type, or publication status were applied. In searching each database, we used a combination of English text and Medical Subject Heading terms, including “home management” and “malaria” and “artemisinin or chloroquine”. A complete search strategy is depicted in the appendix. Bibliographies of screened and selected studies, as well as review articles were manually reviewed to identify any additional relevant studies.

Titles and abstracts of retrieved articles were first screened by one author, and all potentially relevant full texts were screened and evaluated by two authors independently. In the case of any discrepancies, a third author was consulted. Studies were considered eligible for inclusion if they: (1) were before–after studies, observational studies, or randomised controlled trials; (2) pertained to the use of any antimalarial drugs, including artemisinin-based or quinine-based treatments; and (3) involved the delivery of home-based malaria prevention or treatment. Studies were excluded if they were: (1) retrospective studies, case series, case reports, cross-sectional studies, qualitative exploratory research, commentaries, or editorials; (2) studies that did not report separate data for home-based management of malaria; and (3) studies that did not provide clinical or health-related outcomes. Two reviewers independently extracted data using a standardised data abstraction form. Information extracted included study design, study period, participant characteristics, antimalarial drugs for treatment and prevention of malaria, details of home delivery of malaria care, and main study findings. Any differences were discussed and resolved through consensus.

### Outcome measures

Primary outcomes of interest were malaria mortality and all-cause mortality. Secondary outcomes included number of febrile episodes treated with antimalarial drugs, proportion of effective antimalarial treatments (i.e. any antimalarial therapy that consisted of chloroquine plus sulfadoxine–pyrimethamine, quinine, or an artemisinin were deemed to be effective), incidence of clinical malaria episodes, risk of severe malaria, rates of anaemia, parasitaemia, splenomegaly, early treatment failure, late treatment failure, late parasitological failure, sensitivity and specificity for diagnosis of malaria, adherence to antimalarial medications, and adverse drug events.

### Risk of bias assessment

Potential risk of bias was assessed for observational studies based on ROBINS-I tool [23], while randomised controlled trials were judged with the use of a revised Cochrane risk of bias tool (RoB 2.0) [24] and cluster-randomised controlled trials were evaluated using an adapted Cochrane risk of bias tool for cluster-randomised trials (RoB 2.0 CRT) [25].

### Data analysis

Data from each of the studies were extracted and summarised by two authors independently. A standardised data abstraction form in Microsoft Word was used to outline the principal components of each individual study which included study design, study period, sample size, participant characteristics, antimalarial drugs for treatment and prevention of malaria, details of home delivery of malaria care, relevant study outcomes, and main findings. Any discrepancies and disagreements during data extraction were discussed and reviewed among two authors (KP and BC), and a third author (SL) was consulted if consensus was not reached. When comparable quantitative data were reported across multiple studies, we noted the sample sizes, proportions, and frequency statistics to facilitate subsequent pooling of estimates using random-effects models (DerSimonian–Laird method) in order to generate risk ratios (RR) for the dichotomous outcomes. Heterogeneity between studies was evaluated using *I*^2^ statistics and funnel plots were assessed visually for publication bias. All analyses were performed using RevMan version 5.3 (Cochrane Collaboration).

## Results

**Fig. 1:**
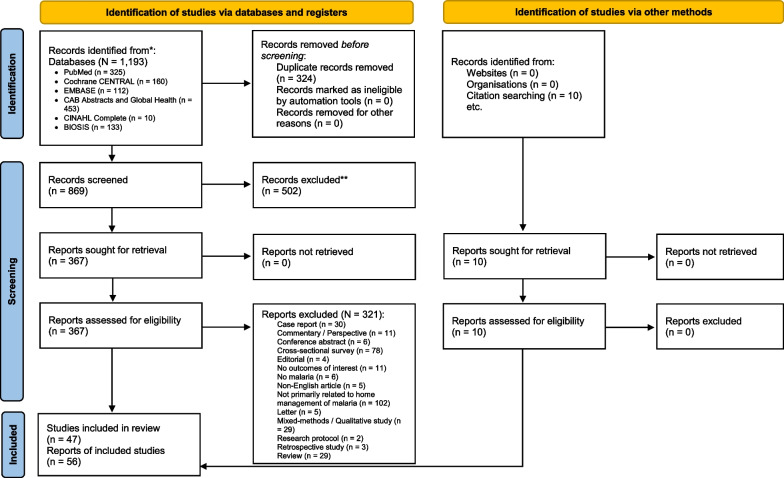
PRISMA 2020 flow diagram for new systematic reviews which included searches of databases, registers and other sources

The literature search yielded 1203 records. After screening of the title and abstract, 377 articles underwent full-text evaluation, of which 56 articles [15, 26–80] representing 47 studies were included in the systematic review (qualitative synthesis) and 40 for quantitative analysis (Fig. 1). Of these, 10 studies were pre–post in design [26–40], 17 were prospective observational studies [15, 41–59], 9 were randomised controlled trials [60–68], and 11 were cluster-randomised controlled trials [69–80]. All studies were conducted in sub-Saharan African countries and were published between 1987 and 2022. They were invariably carried out in remote rural communities (*n* = 40) [15, 26, 27, 29, 31–34, 36–38, 41, 42, 45, 47, 49, 52–61, 64, 66–75, 77, 78, 80], whilst four in peri-urban areas [44, 51, 62, 65], one each in urban areas [76], urban and peri-urban zones [63], and mixed urban and rural areas [50]. The age ranges of participants included in the studies were variable, however, most studies (76.6%) recruited children aged 15 years and younger. The studies had sample sizes ranging from 156 to 34,358 [45, 64], making up a total of 234,002 participants. Antimalarial monotherapy as well as combinations deployed in the home management of malaria were artemether–lumefantrine, chloroquine, chloroquine–sulfadoxine–pyrimethamine, quinine, artesunate–amodiaquine, artesunate–chlorproguanil–dapsone, artesunate–sulfadoxine–pyrimethamine, dihydroartemisinin–piperaquine, artesunate monotherapy, amodiaquine monotherapy, sulfadoxine–pyrimethamine, halofantrine, or *Argemone mexicana* decoction for treating uncomplicated malaria, rectal artesunate for treating severe malaria, and sulfadoxine–pyrimethamine–amodiaquine, sulfadoxine–pyrimethamine–piperaquine, dihydroartemisinin–piperaquine, or pyrimethamine–dapsone as a preventive therapy for malaria. Ten studies reported to utilise rapid tests for malaria diagnosis. Details of individual studies and main findings are summarised in Additional file 1: Table S1.

### Efficacy of home management of malaria

**Fig. 2:**
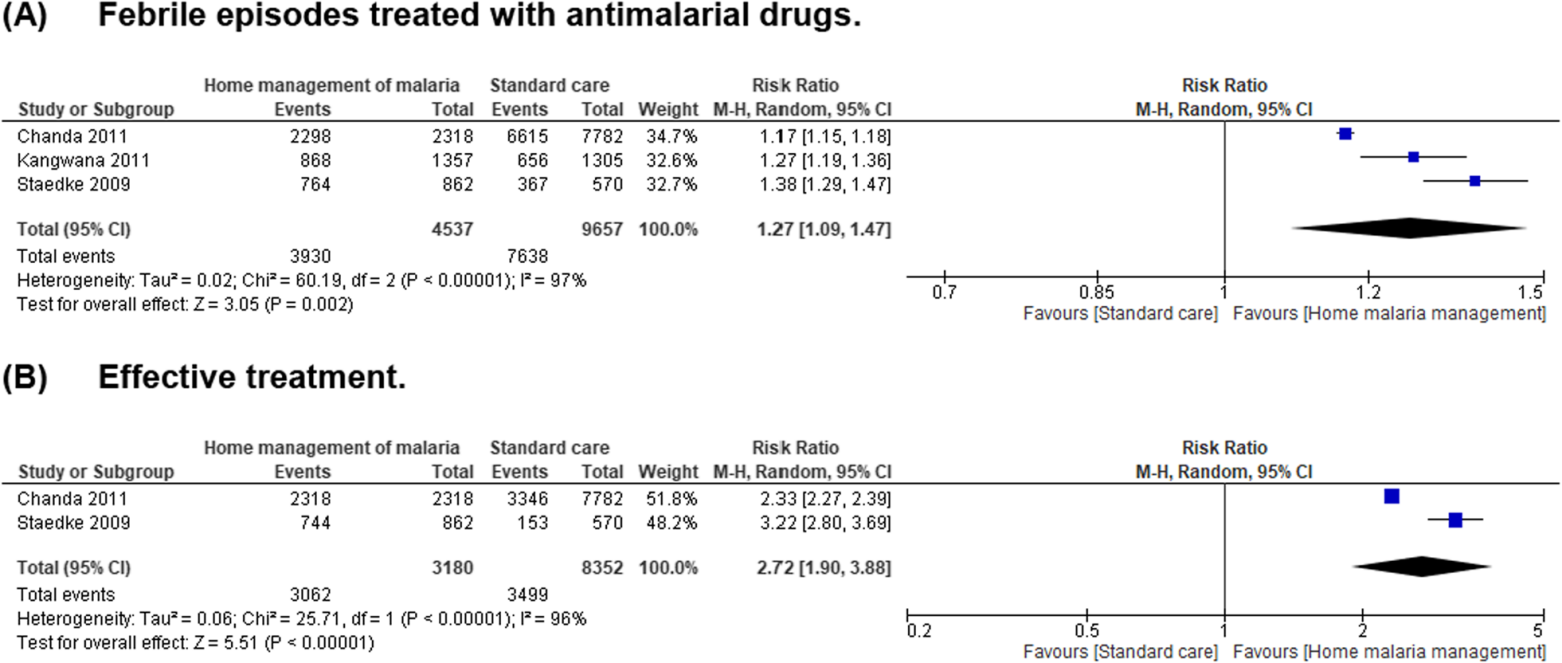
Pooled effect estimates of home management of malaria with artemisinin-based combination therapy compared to standard care. **A** Febrile episodes treated with antimalarial drugs. **B** Effective treatment

**Fig. 3:**
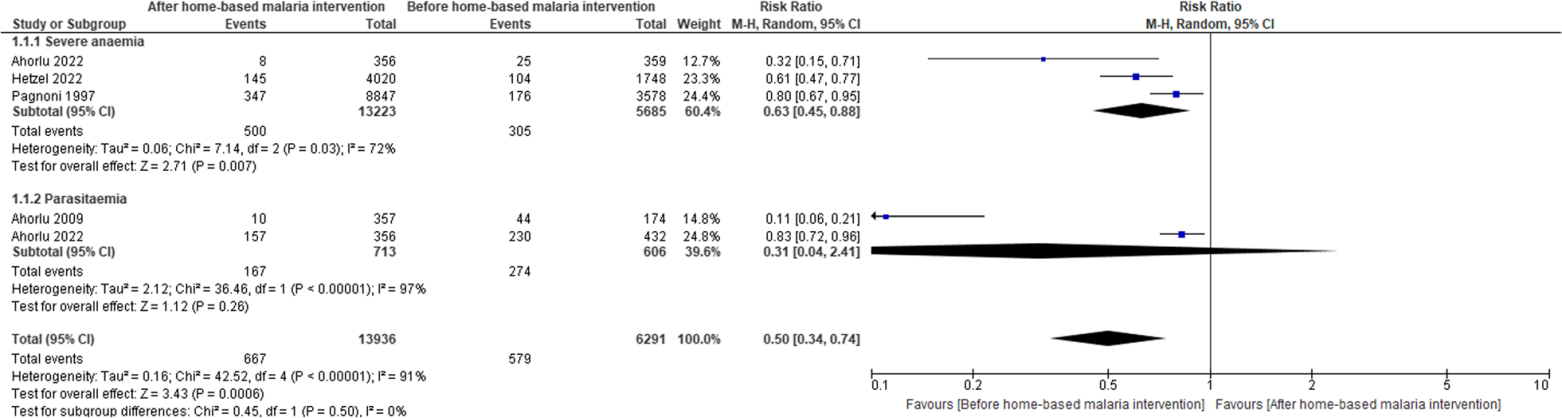
Severe anaemia and parasitaemia before and after implementation of home-based malaria intervention in communities

**Fig. 4:**
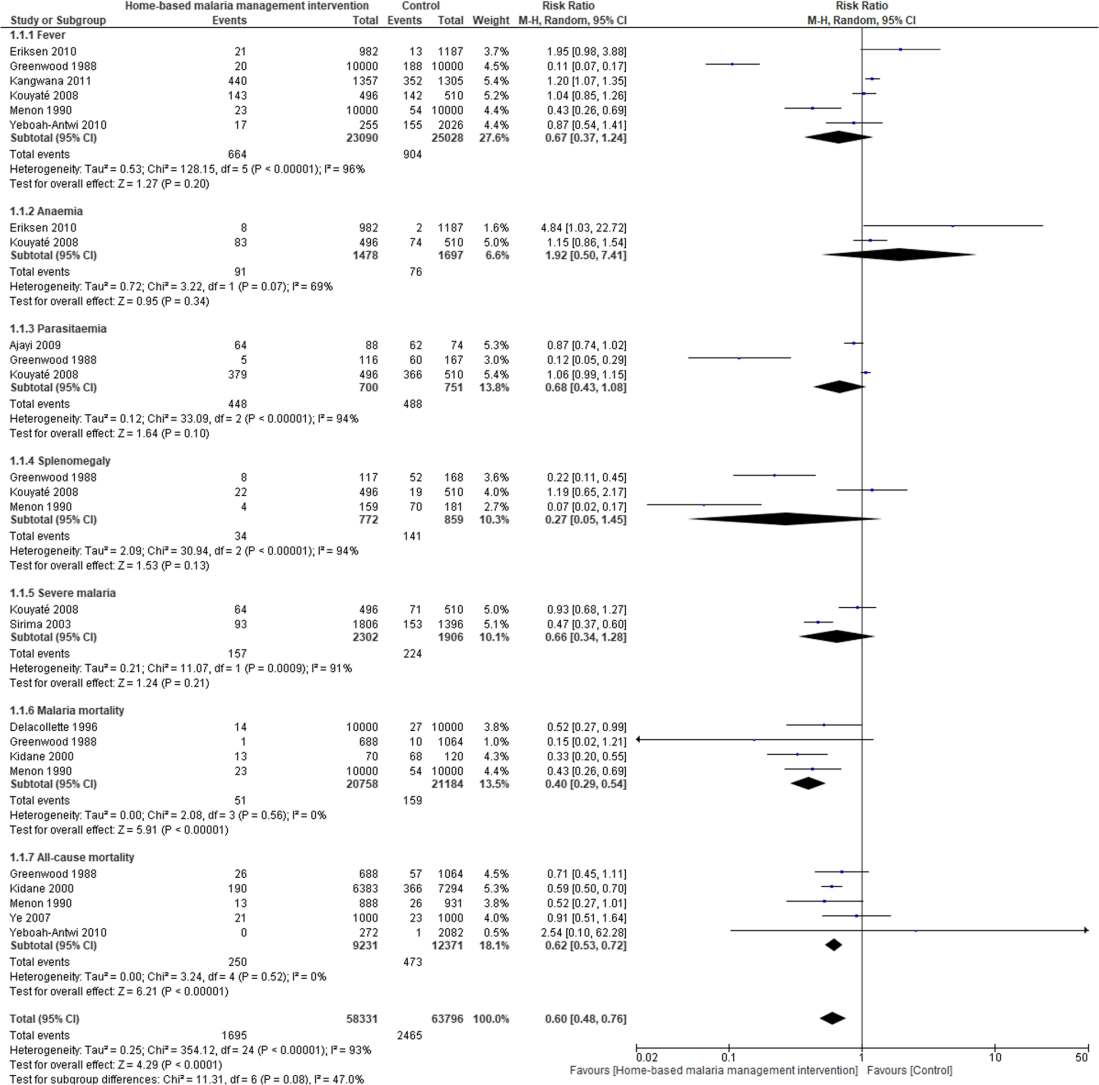
Clinical outcomes of home-based malaria management intervention versus control

**Fig. 5:**
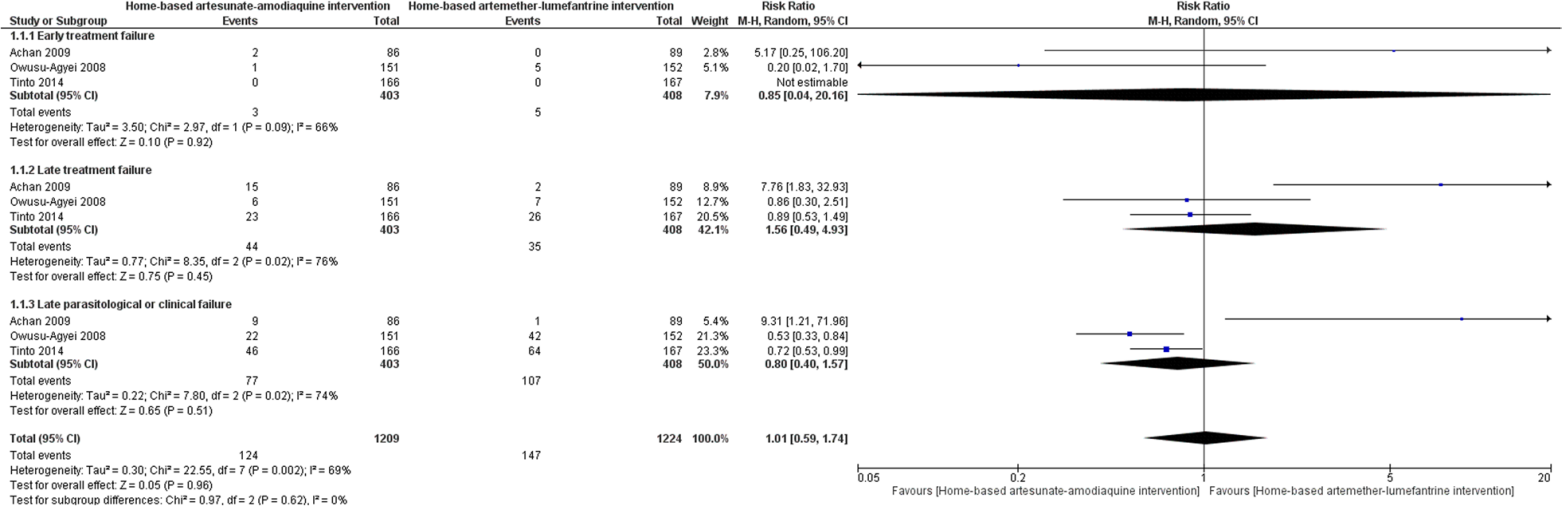
Clinical outcomes of home-based interventions comparing artesunate–amodiaquine with artemether–lumefantrine

Pooled results randomised controlled studies comparing home management of malaria with standard care demonstrated a reduction in the risk of febrile episodes (RR = 1.27, 95% CI = 1.09–1.47, *P* = 0.002, *I*^2^ = 97%) and higher effective rates of antimalarial treatments (RR = 2.72, 95% CI = 1.90–3.88, *P* < 0.00001,* I*^2^ = 96%) in the intervention group (Fig. 2). Among the communities who were provided home management intervention, incidence rates of malaria cases were similar compared with the control group (Table 1A). Combining home-based management with intermittent preventive malaria treatment was associated with a considerably lower incidence risk of malaria than home management intervention that exclusively provided treatment to individuals with febrile illness suggestive of malaria (Table 1B). In a comparison of mortality rates among children under the age of 5 years before and after implementation of home management of malaria, no important difference was noted (Table 2). Contrariwise, the risks of severe anaemia was significantly decreased (RR = 0.63, 95% CI = 0.45–0.88, *P* = 0.007, *I*^2^ = 72%) after the implementation of home-based management of malaria (Fig. 3). Mortality rates due to malaria (RR = 0.40, 95% CI = 0.29–0.54, *P* = 0.00001, *I*^2^ = 0%) and all-cause mortality rates (RR = 0.62, 95% CI = 0.53–0.72, *P* = 0.00001, *I*^2^ = 0%) were similarly reduced among participants receiving home management intervention compared to control group (Fig. 4). However, other outcome including treatment failures, or clinical failure did not differ between groups (Fig. 5). Regarding the diagnostic accuracy of home management of malaria, the relevant studies reported mean sensitivity of 82.9% and specificity of 69.2% (Additional file 1: Table S2). Delivering the home management of malaria intervention also resulted in an average of 94.9% of participants received effective treatment and 90.9% of participants had good adherence to antimalarial regimen (Additional file 1: Table S3).

**Table 1:** Incidence of clinical malaria following implementation of home-based interventions in communities

	Home management of malaria	Control
*A. Treatment of malaria illnesses*		
Francis et al. (2017), Tanzania [33]	281 per 1000 person-year [*N* = 2800]	–
Graz et al. (2010), Mali [61]	0 per 1000 person-year (severe malaria) [*N* = 298]	–
Ratsimbasoa et al. (2012), Madagascar [55]	0.25 per 1000 person-year [*N* = 1073]	–
Staedke et al. (2009), Uganda [76]	7.42 per person-year [*N* = 225]	6.84 per person-year (standard care) [*N* = 212] 9.70 per person-year (clinic cohort) [*N* = 263]
Thiam et al. (2012), Senegal [58]	**2008** 2616 per 100,000 **2009** 1905 per 100,000 (population in 2009: 3,202,760)	**2008** 2339 per 100,000 **2009** 1704 per 100,000 (population in 2009: 1,021,296)
Tiono et al. (2008), Burkina Faso [15]	277 per 1000 (*N* = 7621)	483 per 1000 (*N* = 7605)
Willcox et al. (2011), Mali [68]	310 per 1000 (*N* = 101)	–
Delacollette et al. (1996), Democratic Republic of the Congo [47]	74 per 10,000 (*N* = 13,084) [prevalence] 104 per 10,000 person-weeks [incidence]	109 per 10,000 (*N* = 14,999) [prevalence] 145 per 10,000 person-weeks [incidence]

	Home-based malaria preventive treatment	Home-based malaria management
*B. Intermittent preventive treatment of malaria*		
Sesay et al. (2011), Gambia [66]	44 per 100,000 child-month (*N* = 639)	132 per 100,000 child-month (*N* = 638)
Tine et al. (2011), Senegal [78]	720 per 100,000 child-month (*N* = 500)	3560 per 100,000 child-month (*N* = 500)
Tine et al. (2014), Senegal [79]	491 per 100,000 child-month (*N* = 500)	3440 per 100,000 child-month (*N* = 500)
Ouédraogo et al. (2010), Burkina Faso [64]	3260 per 100,000 child day at risk (*N* = 52)	1070 per 100,000 child day at risk (*N* = 52)

**Table 2:** Childhood mortality rates before and after implementation of home-based malaria intervention in communities

	Before intervention	After intervention
Spencer et al. (1987), Kenya [38–40]	131 per 10,000 (crude death rate) 728 per 10,000 (post-neonatal mortality) 252 per 10,000 (early childhood mortality)	123 per 10,000 (crude death rate) 670 per 10,000 (post-neonatal mortality) 182 per 10,000 (early childhood mortality)
Hetzel et al. (2022), Democratic Republic of the Congo, Nigeria and Uganda [48]	168 per 10,000 (case fatality rate)	439 per 10,000 (case fatality rate)

### Cost of home management of malaria

Five performed an economic evaluation of the home management intervention [37, 45, 51, 58, 76]. The mean cost per home visit was $14.74, which was substantially greater than the cost of follow-up by telephone calls ($0.77) [51]. The cost of delivering home management intervention was $33.83 per child per year, which was associated with lower health care expenditures per participant per year than in the control group [76]. In another study, it was estimated that the annual scale-up costs of home malaria management were $6.73 million for 20% and $11.78 million for 35% utilisation. The cost per case appropriately diagnosed and treated in home-based management was $4.22 as compared to $6.12 for health facility-based management of uncomplicated malaria [45]. Moreover, the total implementation cost of a 4-month home management programme was $12,066, with an average cost of $0.06 per child [37]. The scale-up of home-based management of malaria required $163,424.61 yearly, which was equivalent to $0.80 per person at risk (Additional file 1: Table S4) [58].

### Safety of home management of malaria

17 studies documented data for adverse events [27, 30, 31, 44, 53, 55, 60, 61, 63, 65, 66, 68–70, 75, 77, 79]. There were no significant differences between the groups with respect to adverse event rates [60, 61, 63, 65, 69]. Most studies (64.7%) reported no serious adverse event [27, 30, 31, 55, 60, 61, 63, 66, 70, 75, 77, 79]. Two studies, respectively, identified 1.2% and 4.1% of reported adverse events were serious [44, 53]. Another study depicted severe adverse events only occurring in 3 of 178 participants (1.7%) of home malaria management, of whom two received quinine and one received artemether–lumefantrine [60]. Likewise, during a 3-month follow-up, 3 of 294 participants (1.0%) of home-based management experienced adverse events, namely two deaths in *Argemone mexicana* group and one miscarriage in artemisinin combination therapy group [68]. In a study comparing home malaria management with standard care, one death was recorded in each group [76]. The frequency of adverse events attributable to home malaria management only group was higher than those in combination of home malaria management and preventive therapy group (Additional file 1: Table S5) [77].

Our pooled analysis did not find any discernible differences in the risk of adverse drug events in home management of malaria interventions by use of artesunate–amodiaquine compared with other antimalarial drug combinations (Additional file 1: Fig. S1). Additionally, home-based intermittent preventive treatment had a similar risk of deterioration to severe malaria in comparison with home-based management of malaria-attributable febrile illness (Additional file 1: Fig. S2). When evaluating home management interventions with *Argemone mexicana* decoction versus artesunate–amodiaquine, we did not detect any differences on the risks of severe malaria in children younger than 5 years of age, parasitaemia, and serious adverse events. Nonetheless, provision of *Argemone mexicana* decoction at home increased the need for second-line antimalarial treatment (RR = 3.12, 95% CI = 1.21–8.01, *P* = 0.02, *I*^2^ = 25%) (Additional file 1: Fig. S3).

### Risk of bias

All observational studies had a moderate risk of bias (Additional file 1: Fig. S4) while most randomised controlled trials had some concerns of bias relating to participation in assignment or adhering to intervention and completeness of outcome evaluation and reported result (Additional file 1: Fig. S5). Most cluster-randomised controlled trials were assessed as low risk of bias (Additional file 1: Fig. S6). Examination of the funnel plots for all the meta-analyses indicated that there was no evidence of publication bias.

## Discussion

Home management of malaria is a widely implemented and long-running strategy delivered in malaria-endemic regions across sub-Saharan Africa, where antimalarial drugs are distributed by trained community health workers or community drug distributors, complemented with more recent integration of rapid diagnostic testing through the evolution of community-based programme and point-of-care technologies to minimise overtreatment or antimalarial drug resistance [13, 16]. Nevertheless, the impact of home management intervention on malaria disease burden and patient outcomes remains less understood [8]. Our systematic review and meta-analysis provides the most comprehensive evidence synthesis of the scientific literature on home-based management of malaria for children and adults in communities. It included 47 studies from 17 countries in sub-Saharan Africa, comprising over 230 thousand participants in studies conducted between 1981 and 2020. Considering all health-related outcomes at different time periods that the underlying studies have assessed, our main findings showed that home management of malaria significantly reduced malaria mortality, all-cause mortality, and risks of severe anaemia among patients presenting with symptoms of uncomplicated malaria. The strategy was also associated with better access to treatments for febrile illnesses, effective antimalarial treatments, and good adherence to medications. In terms of home-based preventive treatment, we only detected a significantly lower incidence risk of malaria, but a modest or null effect on severe malaria. The small number of studies on preventive malaria treatment precludes us from quantifying its impact on other clinical outcomes.

Our review found that the number of studies performed in rural populations far exceeds the number of studies in urban populations. Therefore, the collated evidence might not be directly and appropriately translatable to urban communities, including slum households in light of different intensity of malaria transmission [81], geographic proximity and access to health facilities [82], barriers of affordability and symptom recognition, availability of subsidised artemisinin combinations, and communication strategies for urban context [7]. Notably, over 70% of malaria cases in rural areas and 50% of malaria cases in urban areas of Africa are self-treated and formal medical care from health providers is only sought when initial treatment fails [12]. Such health care seeking behaviour underlines the importance of extending the siloed approach of home management intervention beyond rural settings so as to address the plight of populations at risk of malaria living in all types of environment. The scarcity of research in urban and peri-urban settings included in this review mirrors a lack of robust, reliable data to prove the health benefits of home malaria management strategy in urban neighbourhoods [44, 50, 51, 62, 63, 65, 76]. Notwithstanding, the findings generally support the feasibility of providing home management of malaria intervention at the level of households in urban environments.

Several studies (21.3%) utilised rapid diagnostic techniques for the detection of malaria parasites. Timely and accurate techniques for diagnosis are central to effective disease management [83]. Our study showed that rapid diagnostic tests used in the home management intervention had high sensitivity (82.9%), but moderate specificity (69.2%). False positives can arise, thus contributing to inappropriate prescribing or overtreatment with antimalarial agents, with possible undertreatment of alternative causes of febrile illness [84]. Conventional rapid diagnostic tests are unable to detect low density infections under 200 parasites/µL, particularly in non-falciparum infections and Plasmodium falciparum parasites with histidine-rich protein 2 (pfhrp2) and pfhrp3 gene deletions that have been reported to exist in Africa, Asia, and South America [85, 86]. As rapid, point-of-care diagnosis technologies has become a fundamental tool to support home-based malaria management, there is a clear need for reliable, easy-to-use, inexpensive tests that can be conducted at home settings, for instance, CRISPR (clustered regularly interspaced short palindromic repeat)-based malaria diagnostic assay [85] and haemozoin-based malaria diagnostic device [87] to provide robust clinical sensitivity and specificity that help inform decision-making in real time.

Home malaria management tackles barriers to accessing health care services and optimal health for deprived places and socioeconomically disadvantaged, underserved communities. We anticipate that the clinical burden attributable to malaria infections would be alleviated if the intervention is implemented with adequate intensity and over a sufficiently long period or in routine home visits and continuous monitoring of patients infected with malaria via phone calls or physical visits by care providers. The intensity of home management intervention will depend on the number of households in the target population being reached, the frequency of household visitations, diagnostic algorithms being performed, education on malaria preventative measures for patients and their relatives, as well as cascades for seeking medical care at primary health facilities and emergency services. In addition, only five studies in the review evaluated costs of delivering home management intervention [37, 45, 51, 58, 76]. Therefore, we do not have much information on the costs or opportunity costs of home malaria management as compared to other community-based interventions that could be undertaken to mitigate the burden of morbidity and mortality of the infection. The cost-effectiveness of home malaria management is likely to be influenced by factors, such as transmission intensity, environmental changes, and alternative access to quality health care [76, 88]. Exploration and understanding of implementation processes and context along with economic evaluation will provide imperative information to understand what worked, what did not work, and why, as well as inform potential for scale-up and public health policy for malaria control and elimination.

It is noteworthy that the current pooled analysis for the outcomes of malaria mortality and all-cause mortality employed data of studies in rural areas that used chloroquine, which may be now essentially obsolete. Our study also found that home malaria management that involved the use of medicinal herb (*Argemone mexicana* decoction) increased the need for re-treatment with artemisinin-based combination therapies. While *Argemone mexicana* decoction is traditionally used for malaria treatment in several African countries, its clinical effects are less understood [89]. Home malaria management had no effect on child mortality rates, likely to be due to waning of maternally transferred antibodies and lack of innate immune responses to malaria infections [90, 91]. None of the studies in our review ascertained the use of triple artemisinin-based combination therapies, such as dihydroartemisinin–piperaquine–mefloquine or artemether–lumefantrine–amodiaquine for home treatment of malaria. With the increasing treatment failures of conventional artemisinin-based combination therapy, recent studies have revealed that triple artemisinin-based combination therapies are efficacious, well tolerated, and safe for the treatment of uncomplicated falciparum malaria in regions with multidrug-resistant parasites, such as Southeast Asia and South Asia, while prolonging the useful therapeutic lifetime of existing antimalarial drugs that contain lumefantrine [92, 93]. Given that artemisinin-based combination therapies are more complex regimens that would likely compromise treatment adherence and fixed-dose combination has yet to be developed, home management of malaria is a key way to improve patients' adherence to medication regimens.

Our results provide insights on the clinical effects of home malaria management in settings with scarce health care resources. The key strengths of this review include comprehensive database searches, assessment of risk of bias, robust analysis, and the systematic examination of numerous clinical outcomes with the use of artemisinin-based or quinine-based treatments. As a whole, the included studies were judged to have at least moderate overall risk of bias using well-established tools, such as RoB 2.0 and ROBINS-I. The observational studies appeared to provide sound evidence for non-randomised study designs. The main limitations of our research largely pertain to the constraints inherent in the individual studies and data summarised in the meta-analysis. We included only manuscripts published in English. Although we reviewed manuscripts drawn from six online scientific databases, we did not systematically search unpublished data or grey literature. To avoid compositional bias stemming from differences in study-level sociodemographic characteristics in our analysis regardless of geographical region, age, and year. However, due to a paucity of studies published in peer-reviewed journals, we could not identify any relevant research in malaria-endemic countries outside of sub-Saharan Africa, thus precluding us from estimating the health outcomes differentially by race, ethnicity, and geographical location. Moreover, we detected considerable heterogeneity of effect estimates between studies, including febrile episodes treated with antimalarial drugs, effective treatment, parasitaemia, and other clinical outcomes. This could be due several possible explanations, such as study designs, intensity of home management intervention assessed, follow-up durations, lack of standardised data collection tools, and variation between communities and countries in population values and preferences concerning acceptability of home-based approach to managing malaria. Furthermore, a number of randomised controlled trials were performed from the 1980s until mid-2000s. Owing to unavailability of reporting guidelines at the time, these studies may lack details of trial conduct and reporting. Of note, the implementation of home management of malaria intervention may also vary over space and time with different locations scaling up and scaling down at different times. The simplistic approaches that are employed to analyse the data on the large geographic scales might underestimate the true impact or might inaccurately attribute impact to the intervention where there was none. As such, more advanced statistical and modelling methods to measure the overall impact across numerous geographic sites at different times. Spatiotemporal dynamics of clinical outcomes of malaria would be a meaningful approach to identify the impact of home malaria management with artemisinin combination therapy that serves as the front-line treatment against malaria when large surveillance datasets or clinical trials are available. Additional randomised controlled trials evaluating the effects of home malaria intervention, in particular with the use of better logistical support, diagnostic tests, and combination therapies spanning a range of geographical regions are warranted to consolidate the evidence basis for shaping policy solutions to sustainably combat the continuing challenges of malaria elimination [94, 95]. As for urban settings, digital health interventions may yield some encouraging results [96]. Albeit the current evidence suggests that the role of home-based preventive antimalarial treatment is ineffective, it will be of interest to assess its combined effects with vector control and elimination strategies, including use of mobile phone technology for patient and caregiver education [97], new class of insecticide-treated bed nets [98], interventions to address human and local vector population behaviours, or novel, low-carbon house designs [99] plus an annual booster of malaria vaccination [100].

## Conclusions

This updated systematic review and meta-analysis generated evidence to suggest that home malaria management with artemisinin combination therapy led to higher rates of febrile episodes treated with antimalarial drugs and higher effective rate of antimalarial treatments. Our findings also indicated that home malaria management resulted in a good adherence to antimalarial regimen among the patients. Home management with chloroquine was associated with significantly lower incidence of malaria mortality and all-cause mortality, whilst these outcomes were not available with the newer artemisinin combination therapy. A significantly lower incidence risk of malaria was noted by combining home-based management with intermittent preventive malaria treatment. The evidence also reflects that home malaria management intervention is a predominant point of health care access for rural communities. Home malaria management intervention demonstrated a favourable cost-effectiveness profile that supported feasibility for scaling-up in typically high-transmission settings. Reliable diagnostic technologies should be incorporated into home malaria management to allow accurate diagnosis and treatment, thereby improving clinical care. Future randomised controlled trials are needed to build a stronger evidence base and capacity in home-based management of malaria that values diversity in its component designs, participant characteristics, countries, and regions. Effective home treatment algorithms could be implemented in tandem with the use of new antimalarial drugs being developed so as to reduce the massive disease burden and save lives in malaria-endemic regions which are often faced by a grave paucity of resources.

### Supplementary Information

**Additional file 1: Figure S1.** Pooled effect estimates of home management of malaria with artesunate–amodiaquine compared to home management of malaria with other antimalarial drug combinations on risk of experiencing adverse drug events. **Figure S2.** Pooled effect estimates of home-based intermittent preventive treatment compared with home-based management of malaria on risk of developing severe malaria. **Figure S3.** Pooled effect estimates of home management of malaria with *Argemone mexicana* decoction compared to home management of malaria with artesunate–amodiaquine on clinical outcomes. **Figure S4.** Assessment of risk of bias in observational studies based on ROBINS-I tool. **Figure S5.** Assessment of risk of bias in individually randomised controlled trials based on RoB 2.0 tool. **Figure S6.** Assessment of risk of bias in cluster-randomised controlled trials based on RoB 2.0 CRT tool. **Table S1.** Summary of included studies. **Table S2.** Sensitivity and specificity of malaria diagnosis. **Table S3.** Antimalarial treatment following implementation of home-based interventions in communities. **Table S4.** Cost-effectiveness findings from relevant individual studies. Supplementary **Table S5.** Safety findings from individual studies.

## Data Availability

All data generated or analysed during this study are included in this published article and its additional information files.
