# Supplementary material for: The impact of home-based management of malaria on clinical outcomes in sub-Saharan African populations: a systematic review and meta-analysis
Source: Trop Med Health. 2024 Jan 8;52:7. doi: 10.1186/s41182-023-00572-2 (PMC10773121; doi:10.1186/s41182-023-00572-2)
Supplement: Supplementary file 1 — Additional file 1: Figure S1. Pooled effect estimates of home management of malaria with artesunate–amodiaquine compared to home management of malaria with other antimalarial drug combinations on risk of experiencing adverse drug events. Figure S2. Pooled effect estimates of home-based intermittent preventive treatment compared with home-based management of malaria on risk of developing severe malaria. Figure S3. Pooled effect estimates of home management of malaria with Argemone mexicana decoction compared to home management of malaria with artesunate–amodiaquine on clinical outcomes. Figure S4. Assessment of risk of bias in observational studies based on ROBINS-I tool. Figure S5. Assessment of risk of bias in individually randomised controlled trials based on RoB 2.0 tool. Figure S6. Assessment of risk of bias in cluster-randomised controlled trials based on RoB 2.0 CRT tool. Table S1. Summary of included studies. Table S2. Sensitivity and specificity of malaria diagnosis. Table S3. Antimalarial treatment following implementation of home-based interventions in communities. Table S4. Cost-effectiveness findings from relevant individual studies. Supplementary Table S5. Safety findings from individual studies. [file 41182_2023_572_MOESM1_ESM.pdf]

Supplementary Material

Search Strategy

‘home delivery’ OR ‘home management’ OR ‘home-based management’ OR ‘presumptive home treatment’ OR ‘home treatment’ OR ‘treated at home’ OR ‘followed up at home’

AND

‘malaria’ OR malarial infection* OR malaria parasite* OR febrile illness* OR ‘Plasmodium’ OR ‘uncomplicated malaria’ OR ‘uncomplicated Plasmodium falciparum malaria’ OR ‘uncomplicated P falciparum malaria’ OR ‘uncomplicated P. falciparum malaria’

AND

‘artemisinin derivatives’ OR ‘artemisinin antimalarials’ OR ‘artemisinin compounds’ OR ‘artemisinin class’ OR ‘artemisinin’ OR ‘artemisinins’ OR ‘artemisinin-based treatment’ OR ‘artemisinin-based treatments’ OR ‘artemether-lumefantrine’ OR artemisinin combination* OR ‘artemisinin-based combination therapy’ OR ‘artemisinin-based combination therapies’ OR ‘artemether-lumefantrine plus amodiaquine’ OR ‘triple artemisinin-based combination therapy’ OR ‘triple artemisinin-based combination therapies’ OR ‘triple antimalarial combination therapies’ OR ‘dihydroartemisinin-piperaquine’ OR ‘chloroquine’ OR ‘quinine’ OR ‘quinine-based regimen’ OR ‘quinine-based regimens’ OR ‘quinine-based treatment’ OR ‘quinine-based treatments’ OR ‘primaquine’ OR ‘atovaquone-proguanil’ OR ‘artesunate-amodiaquine’ OR ‘artesunate-mefloquine’ OR ‘artesunate-clindamycin’ OR ‘artenimol-piperaquine’ OR ‘artesunate-atovaquone-proguanil’ OR ‘artesunate-sulfadoxine-pyrimethamine’ OR ‘artesunate’ OR ‘mefloquine’ OR ‘atovaquone’ OR ‘proguanil’

Figure S1. Pooled effect estimates of home management of malaria with artesunate-amodiaquine compared to home management of malaria with other antimalarial drug combinations on risk of experiencing adverse drug events.

	Artesunate-amodiaquine		Other antimalarial drugs			Risk Ratio
**Study or Subgroup**	**Events**	**Total**	**Events**	**Total**	**Weight**	**M-H, Random, 95% CI**
Chinbuah 2012	0	863	1	826	0.1%	0.32 [0.01, 7.82]
Niba 2022	83	98	99	115	99.9%	0.98 [0.88, 1.10]
**Total (95% CI)**		**961**		**941**	**100.0%**	**0.98 [0.88, 1.10]**
Total events	83		100			

Heterogeneity: Tau² = 0.00; Chi² = 0.51, df = 1 (P = 0.48); I² = 0%
Test for overall effect: Z = 0.31 (P = 0.76)

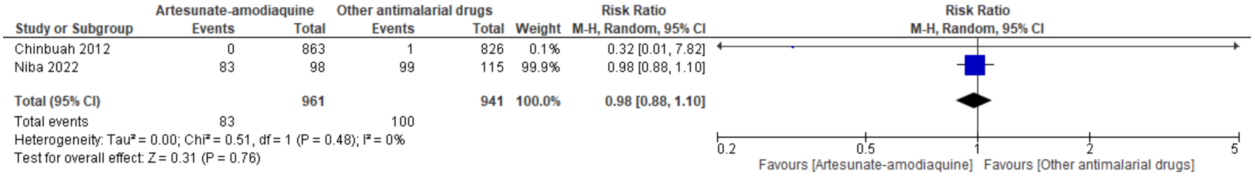

Figure S2. Pooled effect estimates of home-based intermittent preventive treatment compared with home-based management of malaria on risk of developing severe malaria.

	Preventive treatment		Control			Risk Ratio
**Study or Subgroup**	**Events**	**Total**	**Events**	**Total**	**Weight**	**M-H, Random, 95% CI**
Sesay 2011	1	639	3	638	62.1%	0.33 [0.03, 3.19]
Tine 2011	0	500	5	510	37.9%	0.09 [0.01, 1.67]
**Total (95% CI)**		**1139**		**1148**	**100.0%**	**0.20 [0.03, 1.22]**
Total events	1		8			

Heterogeneity: Tau² = 0.00; Chi² = 0.49, df = 1 (P = 0.48); I² = 0%

Test for overall effect: Z = 1.74 (P = 0.08)

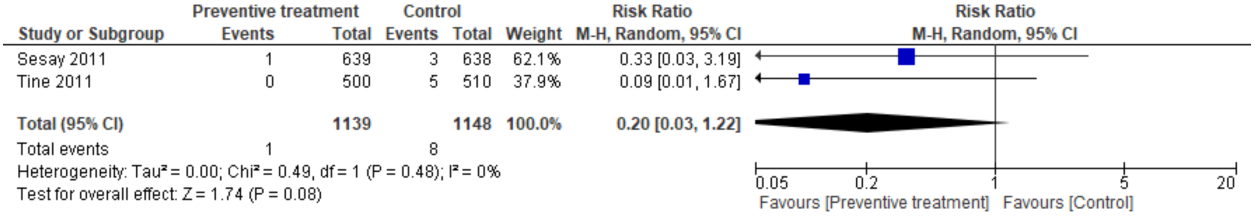

Figure S3. Pooled effect estimates of home management of malaria with *Argemone mexicana* decoction compared to home management of malaria with artesunate-amodiaquine on clinical outcomes.

(A) Severe malaria in children aged ≤5 years

	Argemone mexicana decoction		Artesunate-amodiaquine			Risk Ratio
**Study or Subgroup**	**Events**	**Total**	**Events**	**Total**	**Weight**	**M-H, Random, 95% CI**
Graz 2010	4	197	2	101	61.4%	1.03 [0.19, 5.50]
Willcox 2011	5	102	1	52	38.6%	2.55 [0.31, 21.26]
**Total (95% CI)**		**299**		**153**	**100.0%**	**1.46 [0.39, 5.44]**
Total events	9		3			
Heterogeneity: Tau² = 0.00; Chi² = 0.44, df = 1 (P = 0.51); I² = 0%						
Test for overall effect: Z = 0.56 (P = 0.58)						

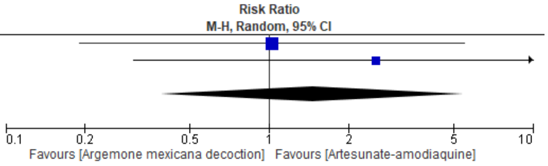

(B) Parasitaemia

	Argemone mexicana decoction		Artesunate-amodiaquine			Risk Ratio
**Study or Subgroup**	**Events**	**Total**	**Events**	**Total**	**Weight**	**M-H, Random, 95% CI**
Graz 2010	25	197	10	101	7.9%	1.28 [0.64, 2.56]
Willcox 2011	116	193	58	101	92.1%	1.05 [0.85, 1.28]
**Total (95% CI)**		**390**		**202**	**100.0%**	**1.06 [0.87, 1.29]**
Total events	141		68			
Heterogeneity: Tau² = 0.00; Chi² = 0.33, df = 1 (P = 0.57); I² = 0%						
Test for overall effect: Z = 0.62 (P = 0.54)						

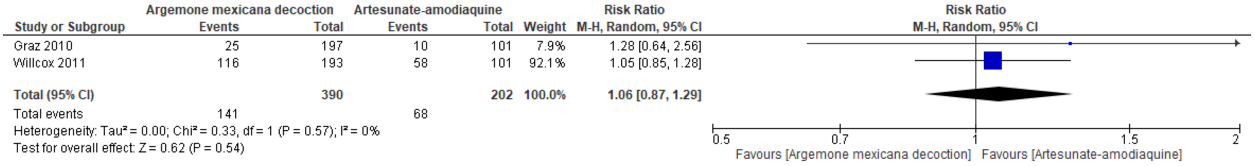

(C) Need for second-line antimalarial treatment

	Argemone mexicana decoction		Artesunate-amodiaquine			Risk Ratio
**Study or Subgroup**	**Events**	**Total**	**Events**	**Total**	**Weight**	**M-H, Random, 95% CI**
Graz 2010	23	197	2	101	35.4%	5.90 [1.42, 24.51]
Willcox 2011	21	193	5	101	64.6%	2.20 [0.85, 5.66]
**Total (95% CI)**		**390**		**202**	**100.0%**	**3.12 [1.21, 8.01]**
Total events	44		7			
Heterogeneity: Tau² = 0.13; Chi² = 1.33, df = 1 (P = 0.25); I² = 25%						
Test for overall effect: Z = 2.36 (P = 0.02)						

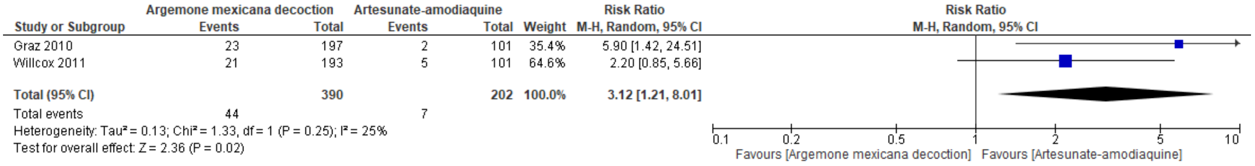

(D) Serious adverse events

	Argemone mexicana decoction		Artesunate-amodiaquine			Risk Ratio
**Study or Subgroup**	**Events**	**Total**	**Events**	**Total**	**Weight**	**M-H, Random, 95% CI**
Graz 2010	0	197	0	101		Not estimable
Willcox 2011	2	193	1	101	100.0%	1.05 [0.10, 11.40]
**Total (95% CI)**		**390**		**202**	**100.0%**	**1.05 [0.10, 11.40]**
Total events	2		1			
Heterogeneity: Not applicable						
Test for overall effect: Z = 0.04 (P = 0.97)						

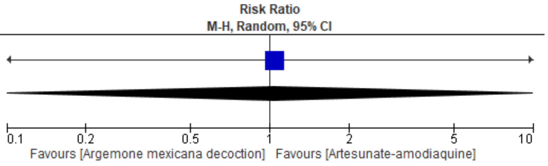

Figure S4. Assessment of risk of bias in observational studies based on ROBINS-I tool.

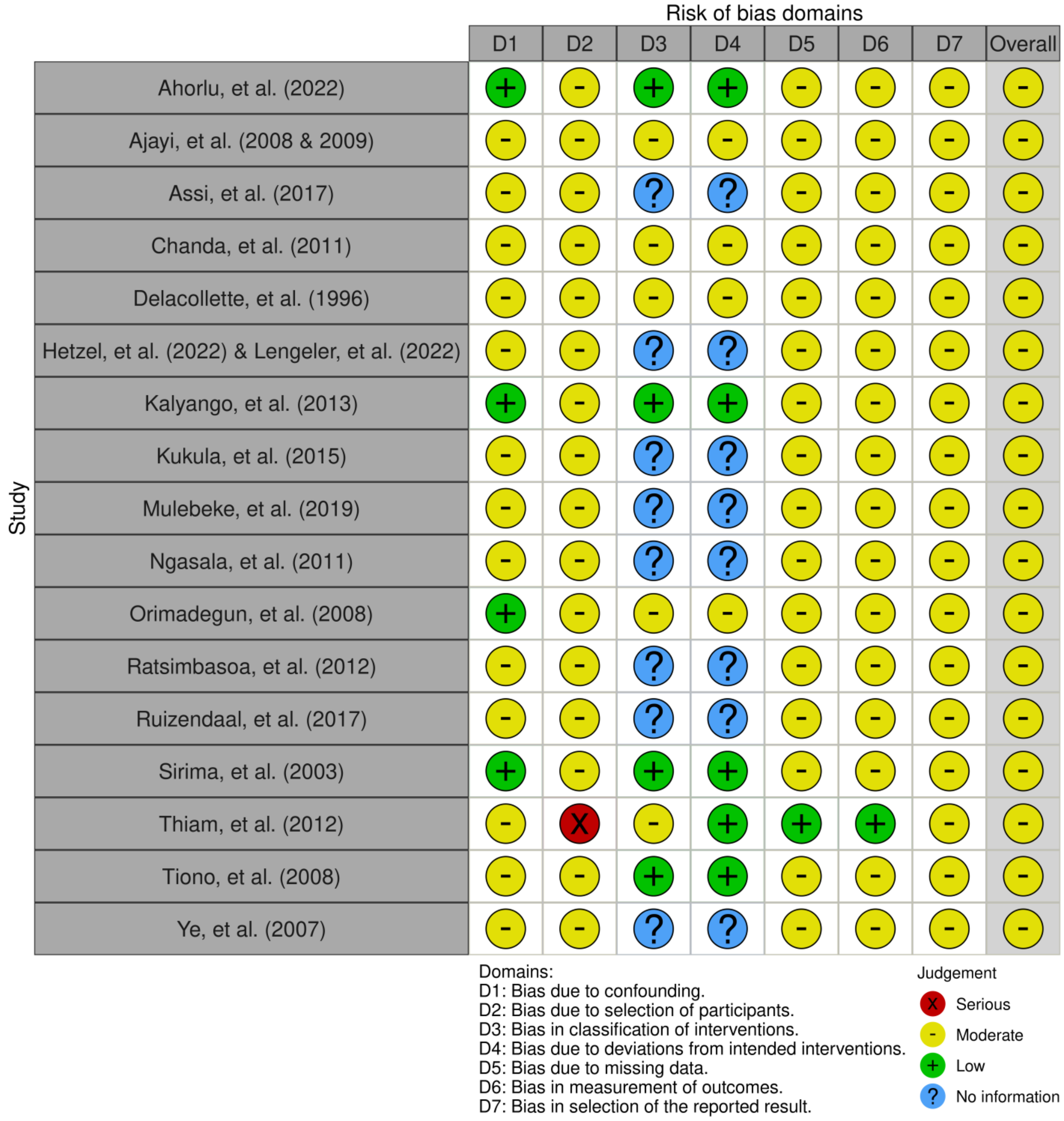

Figure S5. Assessment of risk of bias in individually randomised controlled trials based on RoB 2.0 tool.

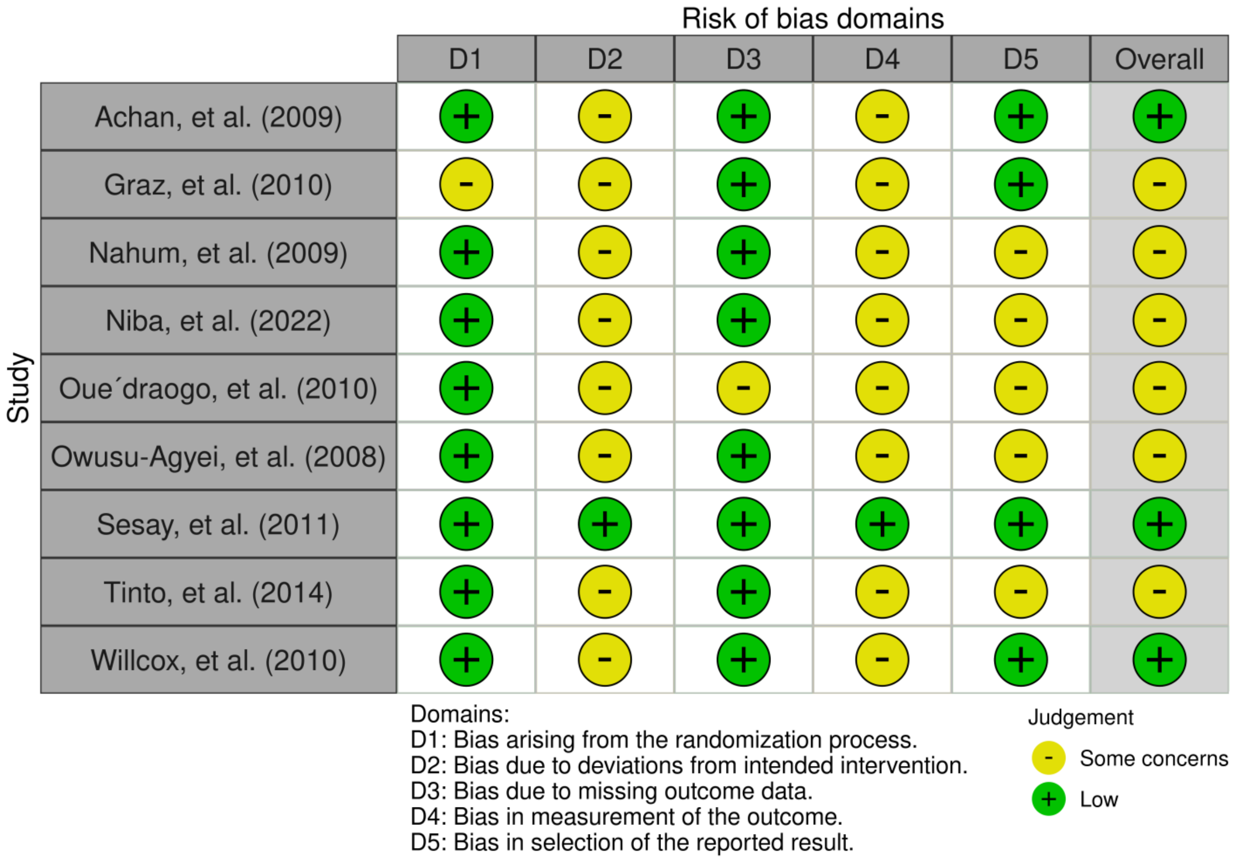

Figure S6. Assessment of risk of bias in cluster-randomised controlled trials based on RoB 2.0 CRT tool.

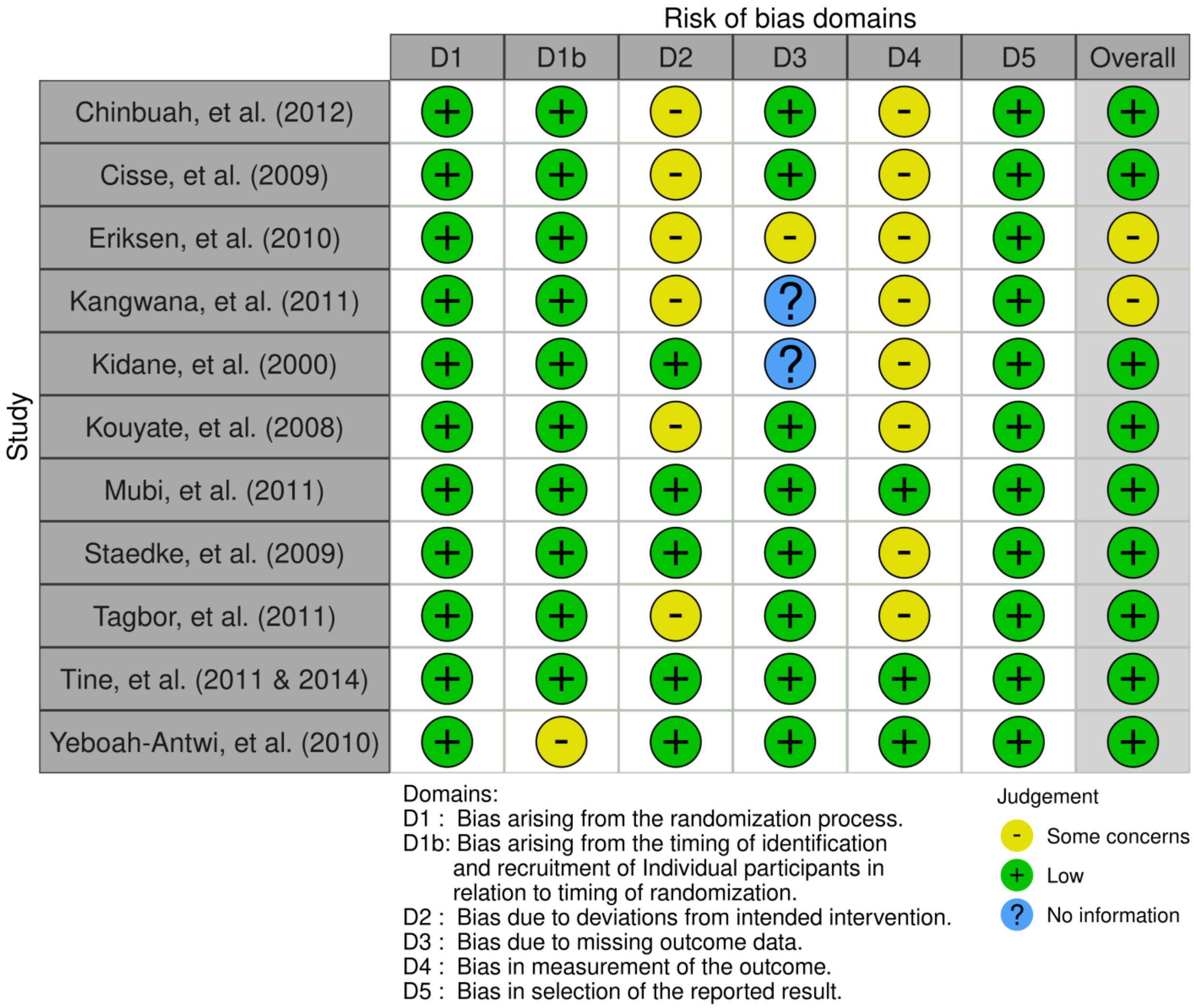

Table S1. Summary of included studies.

Study (year), country	Study population	Implementation period	Antimalarial drugs	Home-based care delivery	Main findings
**Before-after (pre-post) studies**					
Adeoti, et al. (2020), Nigeria [26] [Rural]	Children under 5 years of age with febrile illness (N=1,646)	December 2013 to October 2015	Artemether-lumefantrine (uncomplicated malaria) or rectal artesunate (severe malaria)	Community health workers recognised early signs and symptoms of malaria, performed rapid diagnostic testing, administered treatment, and counselled caregivers following treatment initiation.	Of the 1,646 children treated, 98.5% received the correct treatment based on their rapid diagnostic test results.
Ahorlu, et al. (2009 & 2011), Ghana [27, 28] [Rural]	Children aged 6 to 60 months with suspected febrile malaria (N=365)	July 2007 to March 2009	Artesunate-amodiaquine	Community assistants delivered intermittent preventive treatment of malaria every 4 months with direct observation	Overall prevalence of malaria infection decreased from 25.3% at baseline to 1.0% after 2 years. Marked reduction in the parasite

				and follow-up on Day 1, 2, and 3 after treatment.	prevalence was shown in home delivery of intermittent preventive treatment. A significant reduction was observed in the proportions of febrile children, decreasing from 13.8% at baseline to 2.1% after 2 years. There was no report of serious adverse drug events. No caretaker had lost a child during the 24-month of research implementation.
Ajayi, et al. (2008), Ghana, Nigeria, and	Children aged 6 to 59 months with a complaint of fever	-	Artesunate-amodiaquine (Ghana) or artemether-	In Ghana and Nigeria, caregivers were given antimalarial drugs and administration	Of 1,740 children enrolled, 68.3% had patent parasitaemia prior to treatment. The cure rate

Uganda [29, 30] [Rural]	(N=1,740)		lumefantrine (Nigeria and Uganda)	instructions, following which caregivers treated their children at home without supervision. In Uganda, the first dose of antimalarial drugs was administered to the child in the presence of a community medicine distributor and subsequent doses were administered unsupervised. Home visits were conducted to remind caregivers to administer the medication.	was 97.2% in Uganda, 90.9% in Nigeria, and 91.4% in Ghana. 94.3% of children were reported to comply with correct treatment in terms of dosage and duration. No serious adverse events were reported.
Chinbuah, et	Children aged 6	August 2004 to	Artemether-	Community-based agents	All 204 children aged

al. (2006), Ghana [31] [Rural]	to 59 months with uncomplicated malaria or fever (N=764)	May 2005	lumefantrine	examined signs and symptoms, dispensed artemisinin-based combination therapy, and counselled caregivers on case management and referral.	between 6 and 35 months and 103 of 118 children (87.3%) aged between 36 and 59 months received the correct drug dose from their caregivers. Overall medication adherence was achieved in 92.5% of the children. The intervention improved the time of seeking treatment by 1 day. Of 5 adverse reactions reported, only 1 was associated with the study medication. There was no serious adverse drug event.
Elmardi, et al. (2009),	Individuals of all age groups with	May 2007 to January 2008	Artesunate plus sulfadoxine-	Malaria control assistants conducted rapid	Home-based management of malaria improved

Sudan [32] [Rural]	malaria (N=23,733)		pyrimethamine (uncomplicated malaria) or rectal artesunate (severe malaria)	diagnostic testing, provided treatment, picked up severe malaria and non-malaria cases for referral, and performed stock management.	accessibility to artemisinin-based combination therapy from 25.0% to 64.7% (P=0.004) and treatment seeking behaviour of mothers for their febrile child from 83.3% to 100% (P=0.099). Number of deaths was reduced from 61 to 1 (P<0.001), of which 100% and 0% of the deceased were children under 5 years (P=0.016).
Francis, et al. (2017), Tanzania [33] [Rural]	Individuals of all age groups with febrile illnesses or malaria (N=2,934)	November 2013 to October 2014	Artemether-lumefantrine	Village health workers deployed a mobile phone-based system to follow up patients through daily home visits to give and	Of 860 patients receiving artemether-lumefantrine, 98.2% adhered to treatment. Only 9 patients (1.05%) had malaria

				supervise intake of artemisinin-based combination therapy, monitor treatment outcomes, and collect blood specimens on Day 7.	parasites detected on Day 7 and were retreated. 1 patient with severe febrile illness was referred to hospital.
Greenwood, et al. (1988) & Menon, et al. (1990), Gambia [34, 35] [Rural]	Children aged 3 to 59 months with suspected malaria (N=14,000)	April 1982 to 1987	Chloroquine (presumptive malaria) or pyrimethamine-dapsone (malaria chemoprophylaxis)	Village health workers were trained on how to treat febrile illnesses suggestive of malaria at home. They also provided malaria chemoprophylaxis or placebo to eligible children in the villages every fortnight. They recorded patient medication compliance on	Chloroquine treatment alone had no significant effect on malaria morbidity and mortality. However, combination of treatment with chemoprophylaxis resulted in 49% reduction in mortality and 73% reduction in incidence of clinical malaria.

				registers.	
Nsungwa-Sabiiti, et al. (2007), Uganda [36] [Rural]	Children aged less than 5 years with fever (N=1,085)	August 2002 to September 2004	Chloroquine plus sulfadoxine-pyrimethamine	Drug distributors recognized illness symptoms, provided antimalarial drugs, educated mothers about care seeking and treatment of fever during home visits to the sick child and through village meetings.	Home-based management was associated with 13.5% higher proportion of febrile children completing all treatment steps, translating to 10.4% improvement in community effectiveness of malaria treatment.
Pagnoni, et al. (1997), Burkina Faso [37] [Rural]	Children aged less than 5 years with uncomplicated malaria (N=757)	February 1994 to December 1995	Chloroquine	Mothers and community health workers were presented an algorithm for diagnosis of uncomplicated malaria. Mothers were trained to make decisions to treat,	In the first year of program implementation, the overall proportion of severe malaria cases was lower than the average of 4 preceding years (3.7% vs. 4.9%). In the second year, the

				whilst community health workers supplied drugs for presumptive treatment, provided the child did not require referral.	proportion of severe cases was lower in health facilities with a programme coverage of 50% or more in their catchment area compared with health centres with lower coverage (4.2% vs. 6.1%).
Spencer, et al. (1987), Kenya [38-40] [Rural]	Children aged 9 years or younger with uncomplicated malaria (N=2,040)	1981 to 1983	Chloroquine	Community health workers provided antimalarial treatment.	The intervention had no significant effect on malaria-specific mortality rate in infants and young children, crude birth rate, seropositivity rate to *P. falciparum*, and malaria prevalence.
**Prospective observational studies**					
Ahorlu, et al.	Children under	July 2017 to	Artesunate-	Through biweekly home	The intervention reduced

(2022), Ghana [41] [Rural]	15 years of age (N=460)	July 2018	amodiaquine or artemether-lumefantrine	visits, community-based health volunteers provided timely testing of febrile cases (suspected malaria) using rapid diagnostic tests, promptly treated those with malaria parasites, and referred those who were negative to a health facility for further evaluation.	prevalence of asymptomatic parasitaemia by 33% in 2017 to 44.1% in 2018 after adjusting for age, mean ambient temperature, and insecticide-treated net use (OR=0.67, 95% CI=0.50-0.89, P=0.006) and reduced severe anaemia cases by 67%.
Ajayi, et al. (2008 & 2009), Nigeria [42, 43] [Rural]	Children aged below 10 years with fever (N=611)	March 2002 and October 2005	Chloroquine	Mother trainers were taught to recognise symptoms and signs of malaria, administer treatment for uncomplicated malaria, and refer severe malaria	There was a significant increase in correct use of chloroquine in the intervention group compared to control (OR=0.02, 95% CI=0.00-0.008, P<0.001). Malaria

				and other illnesses to a health centre. They formed a core group of mothers to provide education on malaria diagnosis, as well as treatment and prevention to other caregivers or mothers in the communities.	treatment commenced significantly earlier in the intervention group than those in control (P<0.005). Prevalence of parasitaemia on Day 0 was lower in the intervention group compared to control (72.7% vs. 83.8%, P=0.09).
Assi, et al. (2017), Cote d'Ivoire [44] [Peri-urban]	Individuals of all age groups with suspected uncomplicated malaria (N=12,198)	February 2010 to October 2013	Artesunate-amodiaquine	Community health workers conducted follow-up visits at home to document signs or symptoms, assess patient compliance, detect adverse events, and refer cases to a health centre.	Compliance with treatment was high. Most reported adverse events (93.9%) were of mild or moderate intensity.

Chanda, et al. (2011), Zambia [45, 46]<br>[Rural]	Individuals of all age groups with uncomplicated malaria (N=34,358)	January 2009 to December 2009	Artemether-lumefantrine	Community health workers performed rapid diagnostic testing and provided artemisinin-based combination therapy.	Home management of malaria by community health workers was more cost effective than facility-based management of uncomplicated malaria ($4.22 vs. $6.12). No deaths and severe cases attributable to disease progression were documented. Health workers could ensure the availability of antimalarial drugs.
Delacollette, et al. (1996), Democratic Republic of	Individuals of all age groups with presumptive malaria	February 1985 to July 1987	Chloroquine	Community health workers were trained to provide simple treatment algorithms for fever and	Health care behaviour of the population in the intervention region changed significantly, with over 65%

the Congo [47] [Rural]	(N=28,083)			early recognition and local management of malaria.	of malaria episodes being treated at community level by the end of study period. Prevalence, incidence, crude parasitological index, and high parasitaemia index for malaria declined dramatically in intervention region compared to control.
Hetzel, et al. (2022) & Lengeler, et al. (2022), Democratic Republic of the Congo, Nigeria and Uganda	Children under 5 years of age with a positive malaria test and signs of severe malaria (N=6,286)	April 2018 to December 2020	Rectal artesunate (severe malaria)	Caregivers recognised illness at home. Community health workers diagnosed and administered rectal artesunate. After that, the ill child was immediately referred to a hospital or health facility. A follow-up	In Democratic Republic of the Congo and Nigeria, children who received pre-referral rectal artesunate delivered by community health workers had higher risk of dying by Day 28 compared to those who received neither. However,

[48, 49] [Rural]				home visit after 28 days was conducted by nurses to determine health status.	in Uganda, patients who received rectal artesunate were less likely to die or sick at follow-up.
Kalyango, et al. (2013), Uganda [50] [Mix of urban and rural]	Children aged 4 to 59 months with fever or respiratory symptoms or both (N=1,276)	October 2011 to November 2011	Artemether-lumefantrine or artemether-lumefantrine plus amoxicillin	Community health workers gave treatment and medication administration instructions, visited at home on Day 1 and Day 4 after treatment initiation, and referred severe cases to health facilities.	Medication adherence was similar between the artemether-lumefantrine group and artemether-lumefantrine plus amoxicillin group (99.2% vs. 98.5%, P=0.19).

Kukula, et al. (2015), Ghana [51] [Peri-urban]	Individuals of all age groups who were prescribed artemisinin-based combination therapy for suspected or confirmed uncomplicated malaria (N=4,124)	February 2011 to December 2011	Artesunate-amodiaquine, artemether-lumefantrine, dihydroartemisinin-piperaquine, artesunate monotherapy, amodiaquine monotherapy, or sulfadoxine-pyrimethamine	Field workers went to patients' homes using motorbikes to follow up on adverse events if the patients did not have phone numbers or could not be reached via phone.	27.4 % of patients reported at least an adverse event. Distance travelled for home visits ranged from 0.65 to 62 kilometres, costing \$0.20 to \$79.70. Cost of home visits was approximately 2 times higher than the cost of telephone call follow-up.
Mulebeke, et al. (2019), Uganda [52] [Rural]	Individuals of all age groups (N=14,468)	November 2016 to December 2018	Dihydroartemisinin-piperaquine or artemether-lumefantrine	Community health workers conducted door-to-door mass drug administration delivery, follow-up, and monitored for drug administration	Fixed site distribution and door-to-door follow-up resulted in a high population coverage (≥80%) and facilitated community participation and adherence

				and adherence.	(≥80%) to mass drug administration.
Ngasala, et al. (2011), Tanzania [53] [Rural]	Children aged 3 to 59 month with uncomplicated fever and a positive rapid malaria diagnostic test (N=244)	March 2007 to August 2007	Artemether-lumefantrine	Community health workers recognised symptoms, performed rapid diagnostic testing, obtained blood specimens, provided treatment, and referred patients. Home visits were conducted if patients did not attend scheduled follow-ups.	On Day 7, all patients were not feverish. On Day 42, 141 of 241 patients (58.5%) had recurrent parasitaemia. The cure rates on Day 14, 28 and 42 were 97.9%, 95.1%, and 93.0% respectively. Plasma lumefantrine concentration was significantly higher in patients with reinfections (205 ng/ml) or no parasite reappearance (217 ng/ml) as compared to recrudescence (97 ng/ml, P=0.046). No mortality

					occurred. There were 2 serious adverse events, but unrelated to antimalarial treatment.
Orimadegun, et al. (2008), Nigeria [54] [Rural]	Children aged 5 months to 10 years with fever, malaria parasitaemia, and clinical features of severe malarial anaemia and cerebral malaria (N=268)	-	Chloroquine, amodiaquine, sulfadoxine-pyrimethamine, quinine, halofantrine, or artesunate	Mothers and caregivers were trained to recognize symptoms associated with uncomplicated malaria and provided initial treatment at home before seeking additional care.	Treatment with chloroquine at home was associated with 1.63-fold higher risk of developing cerebral malaria compared to no treatment, while 4-fold increased risk of mortality compared to other antimalarial drugs.

Ratsimbasoa, et al. (2012), Madagascar [55] [Rural]	Children aged 2 to 59 months with suspected malaria (N=1,073)	2008 to 2009	Artesunate-amodiaquine	Community health workers assessed signs and symptoms, provided information to parents, managed antimalarial treatment, obtained blood specimens, and conducted scheduled home visits on Day 3, 28, and 42.	Overall, 90.0% of the children were compliant in terms of medication dosage and duration. Treatment failure rates were 1.6% on Day 28 and 2.1% on Day 42. There were 2 reports of death following presumed severe pneumonia.
Ruizendaal, et al. (2017), Burkina Faso [56] [Rural]	Pregnant women without a known sensitivity to sulfonamides (N=861)	March 2014 to January 2016	Artemether-lumefantrine	Community health workers Were trained to identify malaria symptoms and danger signs and screen for malaria. They paid monthly visits to pregnant women in their second	79.1% of participants with malaria received artemether-lumefantrine treatment from community health workers. Adherence to drug regimen was 96.5%.

				and third trimester until delivery. At each home visit, community health workers performed a rapid diagnostic test, recorded results and treatment, collected blood specimens, and administered antimalarial drugs in positive cases. Referral to a health centre was done for serious cases.	
Sirima, et al. (2003), Burkina Faso [57] [Rural]	Children aged less than 6 years with fever (N=3,202)	1998 to 1999	Chloroquine	Mothers were trained to recognise symptoms of uncomplicated malaria and situations warranting referral to a health centre	Overall risk of developing severe malaria was 7.7%. The risk varied by age and was lower in children treated with pre-packaged

				and use pre-packaged antimalarial drugs. Community health workers and village opinion leaders acted as intermediaries between health services and members of the community and offered advice on treatment and referral.	antimalarial drugs compared with untreated children (RR=0.47, 95% CI=0.37-0.60, P<0.0001). Highest risk was observed in the second 6 months of life.
Thiam, et al. (2012), Senegal [58] [Rural]	Individuals of all ages with suspected malaria (N=12,582)	July 2009 to May 2010	Artesunate-amodiaquine	Home care providers performed rapid diagnostic testing, gave antimalarial treatment, and referred patients to a health facility.	In regions practising home-based management of malaria, total deaths per 100,000 and deaths ascribable to malaria per 100,000 were significantly reduced by 15.4% and

					62.5% respectively, whilst no difference was observed in control regions.
Tiono, et al. (2008), Burkina Faso [15] [Rural]	Children aged less than 5 years with uncomplicated malaria (N=15,226)	May 2006 to October 2006	Artemether-lumefantrine	Community health workers or key opinion leaders recognised symptoms, provided treatment, and referred severe cases to a health facility.	Malaria accounted for 87.4% of all-cause episodes of disease in the home-based management group and 34.1% in the control ($P<0.0001$). 90% of malaria cases in the home-based management group had been treated by community health workers or key opinion leaders at the community level.
Ye, et al. (2007), Burkina Faso	Children aged less than 5 years with fever	December 2003 to November 2004	Chloroquine	Trained non-medical staff conducted home visits every week, diagnosed	Mortality rates were similar between intervention and control groups. 14 of 15

[59] [Rural]	(N=867)			for fever, provided treatment, performed finger prick blood tests, and referred unimproved cases to a health facility.	deaths (93.3%) occurred in children less than 3 years of age, indicating younger children were at increased risk.
**Randomised controlled trials**					
Achan, et al. (2009), Uganda [60] [Rural]	Children aged 6 to 59 months with uncomplicated malaria (N=175)	September 2007 to April 2008	Quinine or artemether-lumefantrine	Nurses supervised administration of the first dose. Caregivers were counselled about the necessity of complying with the full treatment course, potential side effects, and how to administer subsequent doses at home. Nurse visited the patients' home after treatment was	Cure rates were 96% for the artemether-lumefantrine group and 64% for the quinine group (Hazard ratio=10.7, 95% CI=3.3-35.5, P=0.001). In quinine group, 69% of parasitological failures were due to recrudescence, whilst none in the artemether-lumefantrine group. Adverse

				scheduled to be completed.	events did not differ between both groups. Non-adherence to treatment was higher in the quinine group than artemether-lumefantrine group (55% vs. 17%, P=0.001).
Graz, et al. (2010), Mali [61] [Rural]	Individuals with presumed uncomplicated malaria (N=301)	July 2006 to December 2006	*Argemone mexicana* decoction, artesunate-amodiaquine, or intramuscular injection of artemether	Village health workers screened patients, provided treatment, followed up patients on Day 3, 7, 14, and 28, took blood films, and advised patients to return to the health centre if there was a deterioration or recurrence of symptoms. Home visits were	Medication adherence rate was significantly higher in the artesunate-amodiaquine group than the *Argemone mexicana* group (98.0% vs. 75.1%, P<0.001). On Day 14, 100% of patients receiving artesunate-amodiaquine achieved adequate clinical response compared with 65.7% on

				conducted if patients did not return for follow-up.	*Argemone mexicana* decoction (P=0.019). Over 28 days, the proportion of patients with parasitaemia was significantly lower in the artesunate-amodiaquine group (21-49% vs. 63-76%, P<0.001). Proportion of patients requiring second-line treatment was lower among patients in the artesunate-amodiaquine group (5.0% vs. 10.7%).
Nahum, et al. (2009), Benin [62] [Peri-urban]	Children with fever, a *P. falciparum* mono-infection, and without	July 2003 and January 2005	Chloroquine, sulfadoxine-pyrimethamine, or sulfadoxine-pyrimethamine-	Physicians visited participants at home twice a week, did physical examinations, checked their axillary temperature,	Rate of clinical failure at Day 90 was significantly lower in the sulfadoxine-pyrimethamine-artesunate group (2.7%)

	severe malaria (N=237)		artesunate	collected blood samples for microscopic detection and genotyping.	compared to chloroquine (41.4%) or sulfadoxine-pyrimethamine (38.2%) groups (P<0.001).
Niba, et al. (2022), Cameroon [63] [Urban and peri-urban]	Children aged 6 months to 10 years with uncomplicated malaria (N=242)	May 2019 to April 2020	Artesunate-amodiaquine or artemether-lumefantrine	Community health workers supervised first dose intake, advised on time and mode of administration, and ensured patients' compliance with follow-up schedules.	Medication adherence rates were similar between artesunate-amodiaquine and artemether-lumefantrine groups (99.0% vs. 99.1%, P=1.000). Cure rates on Day 28 were similar (96.9% vs. 95.5%, P=0.797). Mild to moderate adverse events were also similar in both groups (84.7% vs. 86.1%, P=0.774).
Ouédraogo,	Children aged 6	-	Sulfadoxine-	Field workers visited	Proportion of participants

et al. (2010), Burkina Faso [64] [Rural]	to 59 months (N=156)		pyrimethamine or artemether-lumefantrine	participants twice a week at home to examine body temperature and the presence of malaria symptoms, conduct brief physical examination, take blood smears if there was fever, provided antimalarial treatment, monitored administration of doses, and referred severe episodes to a health facility.	who did not develop clinical malaria was lower in the artemether-lumefantrine group (9.6%) compared to sulfadoxine-pyrimethamine (28.8%) and control groups (69.2%, P<0.001). Overall incidence of malaria episodes was higher in groups of artemether-lumefantrine (45.7 per 1,000 child days-at-risk) and sulfadoxine-pyrimethamine (32.6 per 1,000 child days-at-risk) compared to control (10.7 per 1,000 child days-at-risk; P<0.001).
Owusu-	Children aged 6	June 2005 to	Artemether-	Nurses supervised first	There were no significant

Agyei, et al. (2008), Ghana [65] [Peri-urban]	months to 10 years with a history of fever (N=534)	May 2006	lumefantrine, artesunate-amodiaquine, or artesunate-chlorproguanil-dapsone	dose intake and field workers supervised administration of subsequent doses. Field workers conducted home visits on Day 1, 2, 3, 7, 14, and 28 after treatment to solicit adverse events. They also obtained finger prick blood samples for parasitological observations, haematological measurements, and biochemical assessments.	differences in rate of treatment failure between the groups. Parasitological and clinical failure on Day 28 for reinfections was significantly lower in the artesunate-amodiaquine group (14.6%) compared to artemether-lumefantrine (27.6%) or artesunate-chlorproguanil-dapsone (28.1%) groups (P=0.005). Incidence of adverse events was comparable between the groups.
Sesay, et al. (2011),	Children aged 6 to 59 months	May 2008 to January 2009	Amodiaquine plus sulfadoxine-	Village health workers diagnosed malaria cases,	Incidence rates of malaria were similar between

Gambia [66] [Rural]	without clinically significant acute or chronic disease (N=1,277)		pyrimethamine or placebo	scheduled for treatment with artemether-lumefantrine, and referred patients to a health centre.	treatment and control groups (0.44 attacks per 1,000 vs. 1.32 per 1,000 child months at risk, P=0.35). Mean haemoglobin levels at the end of the malaria season were also similar (10.2 g/dl vs. 10.3 g/dl, P=0.55). A patient in the placebo arm died of pneumonia.
Tinto, et al. (2014), Burkina Faso [67] [Rural]	Children aged 6 to 59 months weighing 5 kg or more with a *P. falciparum* infection at a density between	September 2008 to January 2010	Artemether-lumefantrine or artesunate-amodiaquine	Nurses educated parents or guardians on how to administer antimalarial drugs at home. If patients did not attend scheduled visits, a standardised history and physical	At Day 28, unadjusted adequate clinical and parasitological response was significantly higher in artesunate-amodiaquine group than artemether-lumefantrine group (58.4%

	2000 and 200,000/µl and haemoglobin >5 g/dl (N=340)			examination would be performed via home visits.	vs. 46.1%, P=0.02), but no difference was noted after adjustment with polymerase chain reaction data. Occurrence of recrudescent infection was similar in both arms.
Willcox, et al. (2011), Mali [68] [Rural]	Individuals of all ages with presumed malaria (N=301)	2006 to 2007	*Argemone mexicana* decoction, artesunate-amodiaquine, sulfadoxine-pyrimethamine, or artemether-lumefantrine	Village health workers screened signs and symptoms, provided treatment, followed up, obtained blood specimens, and conducted home visits.	From Day 29 to Day 84, the number of new episodes of uncomplicated malaria was significantly higher in patients receiving *Argemone mexicana* decoction for more than 7 days as compared with other therapies (0.435 per patient vs. 0.262 per patient, P=0.011). There

					were no significant differences between treatment groups in incidence of severe malaria and anaemia. 3 serious adverse events were documented which included 2 deaths in the decoction group and 1 miscarriage in the artemisinin combination therapy group.
**Cluster-randomised controlled trials**					

Chinbuah, et al. (2012), Ghana [69] [Rural]	Children aged 2 to 59 months with fever (N=12,333)	January 2006 to December 2009	Artesunate-amodiaquine or artesunate-amodiaquine plus amoxicillin	Community health workers assessed for symptoms and clinical signs of malaria, provided drugs for caregivers for treating their child, counselled on drug administration, supervised first dose ingestion, and referred patients to a health centre.	Mortality rates declined by 30% [Rate ratio (RR)=0.70, 95% confidence interval (CI)=0.53-0.92, P=0.011] in artesunate-amodiaquine group and by 44% [RR=0.56, 95% CI=0.41-0.76, P<0.001) in artesunate-amodiaquine plus amoxicillin group compared with control. Of 7 adverse drug reactions reported, only 1 was found to be associated with the study medication.

Cisse, et al. (2009), Senegal [70] [Rural]	Children aged 3 to 59 months of age (N=1,893)	July 2007 to December 2008	Sulfadoxine-pyrimethamine-piperaquine, sulfadoxine-pyrimethamine-amodiaquine, or dihydroartemisinin-piperaquine	Community health workers visited home to administer the first dose of drug treatment, record the child's weights, check the child's health, and refer to a clinic if the child was unwell.	98% of children who received the first dose from a community worker were reported by the mother to have been given the second and third doses at home, most of whom had no difficulty in swallowing the medications. Risks of malaria and parasitaemia were similar in all 3 groups. Piperaquine groups were better tolerated than the sulfadoxine-pyrimethamine-amodiaquine group, with a significantly lower risk of adverse events.
Eriksen, et al.	Children aged 6	April 2004 to	Sulfadoxine-	Health workers were	Decrease in prevalence of

(2010), Tanzania [71] [Rural]	to 59 months with malaria (N=3,884)	May 2005	pyrimethamine	trained in the principles of malaria case management who then trained women leaders to identify fever, treat, supervise antimalarial drug intake, visit the sick child at home on subsequent 2 days, and refer severe cases or other diseases to a health centre.	anaemia was significantly greater in the intervention group (43.9% to 0.8%) compared to the control group (30.8% to 0.17%) (P=0.038). There were no differences in fever prevalence, reported fever episodes, and mean body weight between intervention and control groups.
Kangwana, et al. (2011), Kenya [72] [Rural]	Children aged 3 to 59 months with fever (N=2,749)	March 2009 to May 2010	Artemether-lumefantrine	Retail outlet staff clinically diagnosed and treated malaria, recognised adverse drug reactions, and referred patients to a health facility.	Proportion of children receiving antimalarial treatment was significantly greater in the intervention arm compared to the control. Proportion of

					children receiving antimalarial therapy on the same day or the next day after developing fever was also significantly greater in the intervention group than control group.
Kidane, et al. (2000), Ethiopia [73] [Rural]	Children aged less than 5 years with malaria (N=14,001)	1996 to 1998	Chloroquine	Mother coordinators were trained to teach neighbour-group mothers to identify malaria symptoms, provide appropriate course of antimalarial treatment, share chloroquine properly, and recognise drug-related adverse reactions.	Home treatment intervention resulted in 40.6% reduction in overall under-5 mortality (95% CI=29.2%-50.6%, P<0.003). Of 190 necropsies, only 13 of 70 (18.6%) in the intervention group were consistent with possible malaria compared with 68 of 120 (56.7%) in control.

Kouyaté, et al. (2008), Burkina Faso [74] [Rural]	Children aged 6 to 59 months with fever episodes (N=1,083)	September 2002 to October 2004	Chloroquine	Women group leaders were trained on malaria knowledge and management. They supervised administration of antimalarial drugs, conducted home visits on the second and third day, and referred severe cases to a health centre.	At baseline, more children in control villages received chloroquine treatment at home compared to intervention villages (64% vs. 35%), but an opposite was noted at follow-up (60% vs. 72%). The intervention had no effect on prevalence of anaemia, fever, splenomegaly, clinical malaria, and parasitaemia.
Mubi, et al. (2011), Tanzania [75] [Rural]	Individuals above 3 months of age with fever (N=2,930)	March 2006 to August 2006	Artemether-lumefantrine	Community health workers diagnosed malaria using rapid diagnostic tests, provided treatment, supervised first dose intake, advised	Use of rapid diagnostic tests by community health workers significantly reduced prescription of artemether-lumefantrine by 45% compared to the group

				patients or guardians on how to administer remaining doses of antimalarial drugs, and referred patients with danger signs.	diagnosed by community health workers.
Staedke, et al. (2009), Uganda [76] [Urban]	Children aged 1 to 6 years without serious chronic disease (N=700)	September 2005 to February 2007	Artemether-lumefantrine	Personnel educated households about malaria management based on materials, distributed antimalarial drugs, conducted monthly home visits to deliver the medication.	Children in the home management group received nearly twice as many antimalarial treatments than control group (4.66 per person-year vs. 2.53 per person-year, P<0.0001), and nearly 5 times more treatments than a comparable clinic-based cohort of children (1.03 per person-year, P<0.0001).

					Proportion of participants with parasitaemia at final clinical assessment was lower in the home management group than in controls (2% vs. 10%, P=0.006). 1 child died in each group due to severe pneumonia and presumed respiratory failure.
Tagbor, et al. (2011), Ghana [77] [Rural]	Children aged 3 to 59 months with presumptive malaria (N=1,490)	April 2007 to November 2008	Artesunate-amodiaquine	Community-based drug distributors provided treatment for febrile episodes and were trained to utilise a checklist of signs and symptoms of uncomplicated and	Incidence of presumptive malaria was significantly lower in communities given home management of malaria and intermittent preventive therapy (Protective efficacy: 61.5%, P=0.018). Geometric mean

				complicated malaria to make judgments as to treat or refer, measure and interpret axillary temperatures using digital thermometers, and keep clinical records of children and administer antimalarial drugs based on age.	parasite density was similar in both home management of malaria and combined home management and chemoprevention group. Children in the home management only group reported more adverse events and lower medication compliance compared with children in the combined home management.
Tine, et al. (2011 & 2014), Senegal [78, 79]	Children aged 1 to 10 years (N=1,000)	2010 to 2011	Artemether-lumefantrine (uncomplicated malaria), rectal artesunate (severe	Community health workers performed rapid diagnostic testing, provided prompt treatment with	Incidence of clinical malaria was significantly higher in the home-based malaria management group compared to children who

[Rural]			malaria), or amodiaquine plus sulfadoxine-pyrimethamine	artemether-lumefantrine, followed up treatment, referred severe cases to a health centre, supervised intermittent preventive treatment administration at health hut, and conducted weekly home visits.	received a combination of intermittent preventive treatment and home-based malaria management (35.6 per 100 vs. 7.2 per 100 children-month at risk, P=0.04). Number of malaria episodes, malaria parasitaemia prevalence, and anaemia prevalence were also significantly lower in children receiving combination intervention. 96.4% of patients with uncomplicated malaria had access to artemisinin combination therapy provided by community

					health workers. There was no adverse event noted after using artemisinin combination therapy. Minor to moderate adverse events were reported in 30.0% of children receiving intermittent preventive treatment.
Yeboah-Antwi, et al. (2010), Zambia [80] [Rural]	Children aged 6 months to 5 years with fever (N=3,125)	December 2007 to November 2008	Artemether-lumefantrine	Community health workers managed febrile illness, including testing for malaria using rapid diagnostic tests and provided treatment for malaria.	Proportion of children presenting with a history of fever who received antimalarial drugs in the intervention arm was 27.5% compared to 99.1% in the control arm, suggesting a significant decrease in

					inappropriate use of antimalarials in the intervention group.

Table S2. Sensitivity and specificity of malaria diagnosis.

	Home management of malaria	No intervention
Ajayi, et al. (2008 & 2009), Nigeria [42, 43]	Sensitivity: 78.1%<br>Specificity: 29.2%	82.3%<br>8.3%
Ruizendaal, et al. (2017), Burkina Faso [56]	Sensitivity: 81.5%<br>Specificity: 92.1%	-
Tine, et al. (2014), Senegal [79]	Sensitivity: 89.2%<br>Specificity: 86.2%	-

Table S3. Antimalarial treatment following implementation of home-based interventions in communities.

	**Effective treatment for malaria**	**Patient adherence to medication**
Adeoti, et al. (2020), Nigeria [26]	1,621/1,646 = 98.5%	1,646/1,646 = 100%
Ajayi, et al. (2008), Ghana, Nigeria, and Uganda [30]	Prompt treatment: 14,124/20,563 = 68.7% Correctly treated: 20,063/20,563 = 97.6%	1,096/1,289 = 85.0%
Ajayi, et al. (2008), Ghana, Nigeria, and Uganda [29]	-	230/387 = 59.4%
Chinbuah, et al. (2006), Ghana [31]	335/363 = 92.3%	308/334 = 92.2%
Francis, et al. (2017), Tanzania [33]	-	843/860 = 98.0%
Kalyango, et al. (2013), Uganda [50]	-	582/589 = 98.8%
Niba, et al. (2022), Cameroon [63]	215/217 = 99.1%	-
Ratsimbasoa, et al. (2012), Madagascar [55]	489/543 =90.1%	497/543 = 91.5%
Staedke, et al. (2009), Uganda [76]	Prompt treatment: 477/862 = 55.3%	-

	Correctly treated: 744/862 = 86.3%	
Thiam, et al. (2012), Senegal [58]	4,126/4,270 = 96.6%	
Willcox, et al. (2011), Mali [68]	-	98/101 = 97.0%
Assi, et al. (2017), Cote d'Ivoire [44]	98.6%	14,736/15,161 = 97.2%
Achan, et al. (2009), Uganda [60]	-	89.95%

Table S4. Cost-effectiveness findings from relevant individual studies.

	**Cost**	**Scale-up expenditure**
Kukula, et al. (2015), Ghana [51]	$14.74 per home visit	-
Staedke, et al. (2009), Uganda [76]	$33.83 per person	-
Chanda, et al. (2011), Zambia [45]	$4.22 per case	$11,783,910.70 per year
Pagnoni, et al. (1997), Burkina Faso [37]	$0.06 per person	$36,198 per year
Thiam, et al. (2012), Senegal [58]	$0.80 per person	$163,424.61 per year

Table S5. Safety findings from individual studies.

	**Adverse events**	**Serious adverse events**
Assi, et al. (2017), Cote d'Ivoire [44]	*2,545 cases* Asthaenia, vomiting, somnolence, and malaria.	*105 cases* Malaria, anaemia, gastroenteritis, asthenia, vomiting, pyrexia, and death.
Owusu-Agyei, et al. (2008), Ghana [65]	*533 cases* Unable to suck/drink, fever, runny nose, cough, difficulty in breathing, diarrhoea, vomiting, itching/pruritus, loss of appetite, nausea, abdominal pain, body pain, difficulty in sleeping, joint pain, palpitation, rash, ulcers in mouth/tongue, and yellow eyes.	-
Niba, et al. (2022), Cameroon [63]	*44 cases* Leukocytosis, lymphocytosis, granulocytopenia, lymphocytosis, thrombocytosis, and fever.	-
Achan, et al. (2009), Uganda [60]	*71 cases* Fever, anorexia, cough, diarrhoea, recurrent seizures, repeated vomiting, and rash.	*3 cases*
Chinbuah, et al. (2012), Ghana [69]	*1 case* Itching.	-

Graz, et al. (2010), Mali [61]	Cough, diarrhoea, vomiting, and dizziness.	*0 case*
Ahorlu, et al. (2009), Ghana [27]	Feeling weak.	*0 case*
Ajayi, et al. (2008), Ghana, Nigeria, and Uganda [30]	*371 cases*	*0 case*
Chinbuah, et al. (2006), Ghana [31]	*5 cases*<br>Itching on the neck, dizziness for short spells, fever startling, greenish stool, crying, restlessness, and drowsy.	*0 case*
Cisse, et al. (2009), Senegal [70]	*977 cases*<br>Vomiting, fever, rash, itching, and headache.	*6 cases*<br>Death.
Mubi, et al. (2011), Tanzania [75]	*24 cases*<br>Nausea, weakness, headache, and diarrhoea.	*0 case*
Ratsimbasoa, et al. (2012), Madagascar [55]	Diarrhoea and sleepiness.	*0 case*
Sesay, et al. (2011), Gambia	-	*0 case*

[66]		
Tine, et al. (2014), Senegal [79]	*195 cases*<br>Nausea, vomiting, sleepiness, abdominal pain, diarrhoea, loss of appetite, headache, pruritus, cough, and dizziness.	*0 case*
Tagbor, et al. (2011), Ghana [77]	*157 cases*<br>Dark urine, dizziness, headache, itching, jaundice, nausea, skin rash, sought medical attention, sleeplessness, vomiting, and weakness.	*0 case*
Ngasala, et al. (2011), Tanzania [53]	*162 cases*<br>Fever, cough, and diarrhoea.	*2 cases*<br>Severe malaria.
Willcox, et al. (2011), Mali [68]	Otitis media, urinary infections, sexually transmitted infections, and malnutrition.	*3 cases*<br>Death and miscarriage.
